# Genomic *ALK* alterations in primary and relapsed neuroblastoma

**DOI:** 10.1038/s41416-023-02208-y

**Published:** 2023-02-17

**Authors:** Carolina Rosswog, Jana Fassunke, Angela Ernst, Birgid Schömig-Markiefka, Sabine Merkelbach-Bruse, Christoph Bartenhagen, Maria Cartolano, Sandra Ackermann, Jessica Theissen, Mirjam Blattner-Johnson, Barbara Jones, Kathrin Schramm, Janine Altmüller, Peter Nürnberg, Monika Ortmann, Frank Berthold, Martin Peifer, Reinhard Büttner, Frank Westermann, Johannes H. Schulte, Thorsten Simon, Barbara Hero, Matthias Fischer

**Affiliations:** 1 Department of Experimental Pediatric Oncology, University Children’s Hospital of Cologne, Medical Faculty, University of Cologne, Cologne, Germany; 2 Center for Molecular Medicine Cologne (CMMC), Medical Faculty, University of Cologne, Cologne, Germany; 3 Else Kröner Forschungskolleg Clonal Evolution in Cancer, University Hospital of Cologne, Cologne, Germany; 4 Department of Pediatric Oncology and Hematology, University Children’s Hospital of Cologne, Medical Faculty, University of Cologne, Cologne, Germany; 5 Institute of Pathology, University Hospital of Cologne, Cologne, Germany; 6 Hopp Children’s Cancer Center Heidelberg (KiTZ), Heidelberg, Germany; 7 Division of Pediatric Glioma Research, German Cancer Research Center (DKFZ) and German Cancer Consortium (DKTK), Heidelberg, Germany; 8 Department of Pediatric Oncology, Hematology, Immunology and Pulmonology, Heidelberg University Hospital, Heidelberg, Germany; 9 Cologne Center for Genomics (CCG), University of Cologne, Faculty of Medicine and University Hospital Cologne, Cologne, Germany; 10 Berlin Institute of Health at Charité – Universitätsmedizin Berlin, Core Facility Genomics, Berlin, Germany; 11 Max Delbrück Center for Molecular Medicine in the Helmholtz Association (MDC), Berlin, Germany; 12 Department of Translational Genomics, Medical Faculty, University of Cologne, Cologne, Germany; 13 Division Neuroblastoma Genomics, B087, German Cancer Research Center and Hopp Children´s Cancer Center at the NCT (KiTZ), Heidelberg, Germany; 14 Department of Paediatric Oncology and Haematology, Charité University Medical Centre Berlin, Berlin, Germany

**Keywords:** Paediatric cancer, Cancer genetics

## Abstract

**Background:**

Genomic alterations of the anaplastic lymphoma kinase gene (*ALK*) occur recurrently in neuroblastoma, a pediatric malignancy of the sympathetic nervous system. However, information on their development over time has remained sparse.

**Methods:**

*ALK* alterations were assessed in neuroblastomas at diagnosis and/or relapse from a total of 943 patients, covering all stages of disease. Longitudinal information on diagnostic and relapsed samples from individual patients was available in 101 and 102 cases for mutation and amplification status, respectively.

**Results:**

At diagnosis, *ALK* point mutations occurred in 10.5% of all cases, with highest frequencies in stage 4 patients <18 months. At relapse, *ALK* alteration frequency increased by 70%, both in high-risk and non-high-risk cases. The increase was most likely due to de novo mutations, frequently leading to R1275Q substitutions, which are sensitive to pharmacological ALK inhibition. By contrast, the frequency of *ALK* amplifications did not change over the course of the disease. *ALK* amplifications, but not mutations, were associated with poor patient outcome.

**Conclusions:**

The considerably increased frequency of *ALK* mutations at relapse and their high prevalence in young stage 4 patients suggest surveying the genomic *ALK* status regularly in these patient cohorts, and to evaluate *ALK*-targeted treatment also in intermediate-risk patients.

## Background

Neuroblastoma is the most frequent extracranial solid cancer in childhood [1]. The tumor presents as clinically heterogeneous disease with courses that range from spontaneous regression to fatal progression despite multimodal treatment. Major risk factors for high-risk disease are genetic alterations that lead to telomere maintenance in the tumor cells, such as amplification of the proto-oncogene *MYCN*, genomic rearrangements of the *TERT* locus [2], and activation of the alternative lengthening of telomeres pathway [3]. In the presence of telomere maintenance mechanisms, mutations of genes of the RAS/MAPK pathway are associated with devastating outcome [3]. The most frequent mutations that lead to activation of the RAS/MAPK pathway in neuroblastoma affect the gene that encodes for anaplastic lymphoma kinase (*ALK*). The ALK protein is a receptor tyrosine kinase that is frequently activated in pediatric malignancies, such as anaplastic large cell lymphoma, inflammatory myofibroblastic tumor, rhabdomyosarcoma, and neuroblastoma [4]. In neuroblastoma, *ALK* mutations are mostly activating missense single nucleotide variants (SNV) leading to increased kinase activity by disrupting the auto-inhibited conformation of the kinase [5, 6], thereby promoting tumor growth, proliferation, and migration [7]. *ALK* point mutations have been reported in 6–17% of all cases of sporadic neuroblastoma [8–15]. The vast majority of *ALK* mutations cluster at three hotspot positions within the tyrosine kinase domain (R1275, F1174, and F1245), accounting for about 85% of all *ALK* variants in neuroblastoma [8, 12, 16]. Mutations at position F1174 and F1245 are almost exclusively found in sporadic disease, whereas mutations at R1275 are also present as germline variants in familial cases. Autosomal dominant gain-of-function *ALK* mutations are reported in about 50% of familial neuroblastoma cases [17]. In addition to point mutations, focal genomic amplification of *ALK* has been reported in 2–10% of neuroblastoma [5, 11, 12, 15, 17–19]. Such alterations occur almost always in combination with amplification of *MYCN*, and mutually exclusive with *ALK* point mutations [18], with few exceptions [15]. Patients whose tumors harbor *ALK* amplification have poor outcomes [12]. The prognostic significance of somatic *ALK* SNV, however, has been discussed controversially, as poorer survival has been reported in the subgroups of intermediate and high-risk patients in some studies [12, 15], while others did not find prognostic relevance of *ALK* mutations [11, 19]. In contrast to SNV and amplification, translocation of *ALK* has been reported only occasionally in neuroblastoma [20, 21].

ALK is a tractable target in tumors bearing activating *ALK* alterations, and various ALK inhibitors have been developed. The ALK inhibitors crizotinib and ceritinib have been evaluated in early clinical trials in neuroblastoma and other pediatric malignancies [4, 22, 23], showing promising clinical activity in a fraction of cases. Third-generation ALK inhibitors, such as lorlatinib, may overcome primary resistance in *ALK*-mutated neuroblastoma, and are currently evaluated in clinical trials.

Although the overall frequency of *ALK* alterations in neuroblastoma at diagnosis has been well documented, little is known about their prevalence and prognostic impact in specific patient subgroups, still, and in patients with relapsed or progressive disease in particular. Since ALK inhibitory therapies are primarily considered for patients with relapsed or progressive disease in current treatment strategies, it appears to be essential to gain detailed knowledge of the frequencies and spectrum of *ALK* alterations in this cohort. We, therefore, set out to determine *ALK* alteration frequencies and associations with clinical variables in a large cohort of diagnostic and relapsed neuroblastomas that were obtained from patients treated within successive trials and registries of the Gesellschaft für Pädiatrische Onkologie (GPOH).

## Methods

### Patients

Patients included in this study were diagnosed with neuroblastoma and covered the entire spectrum of the disease. Patients were selected based on availability of tumor *ALK* mutation and/or *ALK* amplification status and corresponding clinical follow-up data. Thereby, reports on *ALK* testing were collected retrospectively. Furthermore, tumor specimens were investigated for the purpose of this study in the lab of experimental pediatric oncology in Cologne to validate *ALK* alterations found previously and to understand their dynamics over the course of disease. Of note, the cohort of samples with *ALK* amplification status was biased towards *MYCN*-amplified tumors, as *ALK* amplification status is routinely assessed in *MYCN*-amplified tumors. Two sub-cohorts had been published previously (*n* = 263 [13] and *n* = 436 [3]). Patients included in this study were diagnosed between 1989 and 2020, and treated according to the successive German trial protocols NB85 (*n* = 1), NB90 (NCT00002802, *n* = 24), NB95-S (NCT00002803, *n* = 2), NB97 (NCT00017225, *n* = 249), NB2004/NB2004-HR (NCT00410631/NCT03042429, *n* = 551), and NB2016-Registry (DRKS00023442, *n* = 97) with informed consent by patients or their legal guardians. The trials were conducted in accordance with the Declaration of Helsinki and Good Clinical Practice and were approved by the institutional ethical review boards of the University of Cologne. Further, a cohort of patients not included in any clinical trial but treated accordingly in Cologne (*n* = 19), was considered for analyses. Additionally, a cohort of patients, registered in the INFORM registry trial was included in this study (Supplementary Table 1). All samples were checked histologically for tumor cell content by an experienced pathologist.

**Fig. 1:**
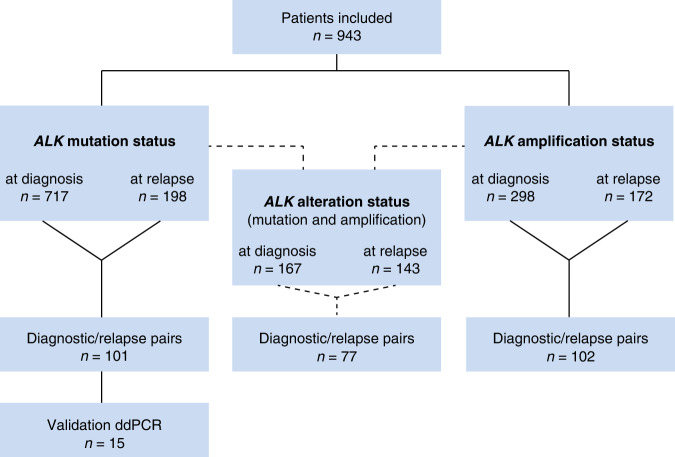
Consort diagram of the study. Flow diagram of patient inclusion in this study. Patients that are listed in the category “*ALK* alteration status” represent the overlap of the cohorts for which *ALK* mutation and for which *ALK* amplification status was available. ddPCR, digital droplet PCR.

In total, 943 patients were included in the study (Fig. 1, Supplementary Table 1). Primary tumor samples were taken at time of initial diagnosis or during first line treatment and relapsed samples were taken at the time of relapse or during relapse treatment. For a sub-cohort we investigated paired samples, taken at diagnosis and at relapse from the same patient. For *ALK* mutation status 717 primary tumors samples and 198 samples taken at relapse were analyzed. Of the primary tumor samples, 352 were derived from high-risk tumors, defined by *MYCN* amplification, or stage 4 disease for patients ≥18 months, and 365 were derived from non-high-risk tumors, defined as localized disease, or stage 4 disease in patients <18 months, each without amplification of *MYCN*. Tumor stages ranged from INSS stage 1–4 and 4S [24, 25]. 172 of the primary tumors showed amplification of *MYCN*, whereas 545 were *MYCN* non-amplified. For *ALK* amplification, we investigated 298 samples taken at diagnosis. From these, 256 were high-risk, and 42 non-high-risk tumors, 186 showed amplification of *MYCN*, whereas 112 were *MYCN* non-amplified.

At relapse, we investigated 198 samples for *ALK* mutations (Fig. 1, Supplementary Table 1). 151 of these were high-risk and 47 were non-high-risk tumors, 64 showed amplification of *MYCN,* and 134 were *MYCN* non-amplified. *ALK* amplification status at relapse was determined in 172 samples. 119 of these were high-risk tumors, 53 were non-high-risk, and 53 showed amplification of *MYCN* whereas 119 were *MYCN* non-amplified.

### Detection of *ALK* alterations

Neuroblastoma samples were analyzed for *ALK* mutations by various methods: dideoxy-sequencing (exons 21–25, *n* = 303), whole-exome or whole-genome sequencing (WES, WGS, *n* = 306), panel next-generation sequencing (panel NGS, *n* = 124) or a combination of at least two of the respective methods (*n* = 185, Supplementary Table 1). For one sample, method for detection of *ALK* mutations remained unknown. Information on germline variants was not available for all samples, thus we did not distinguish between somatic and germline *ALK* mutations. *ALK* amplifications were analyzed by Fluorescence in-situ hybridization (FISH, *n* = 401), low-coverage whole genome sequencing (WGS, n = 16) or by a combination of both methods (*n* = 53, Supplementary Table 1). *ALK* amplification in FISH analyses was defined as >4 copies of *ALK* in relation to copies of chromosome 2 reference. For cases analyzed by low-coverage WGS we defined amplification as copy number of *ALK* locus >10. Samples with ambiguous copy numbers [3–10] in low-coverage WGS were only included when material for validation by FISH was available.

### Fluorescence in situ hybridization (FISH)

FISH was performed using a dual-color probe (Zytolight Spec ALK/2q11 Dual Color, Zytovision) for chromosome 2q11 as reference and a probe specific for the *ALK* gene locus to count the number of *ALK* copies in relation to the number of chromosomes 2 copies as described previously [26].

### DNA extraction

DNA extraction was performed according to the manufacturers’ instructions, using the Puregene Core Kit A (Qiagen) for fresh frozen and Maxwell® 16 FFPE Plus LEV DNA Purification (Promega) for paraffin-embedded tumor material.

### Dideoxy-sequencing

DNA was amplified with primer pairs for *ALK* exons 21–25 (Supplementary Table 2). Dideoxy sequencing was performed as described previously [27]. Sequencing analyses were carried out on the eight capillary electrophoresis system 3500 Genetic Analyzer (Life Technologies).

### Whole exome, whole genome, and low-coverage whole genome sequencing

Whole genome and whole exome sequencing was performed and analyzed as described previously [2, 3]. Cancer cell fractions and copy number values were estimated with Sclust [28]. The inference of mutational clusters and their dynamics over the development of the tumor was performed as described previously [29]. A sub-cohort of samples evaluated in this study was produced and kindly provided by the INFORM program [30, 31].

### Amplicon-based next-generation sequencing

Mutational analysis for low input DNA was performed by next-generation sequencing (NGS) using an Ion AmpliSeq Custom DNA Panel (Thermo Fisher Scientific) and the Ion AmpliSeq Library Kit 2.0 (Thermo Fisher Scientific) according to the Ion AmpliSeq Library Preparation User Guide (Thermo Fisher Scientific). After multiplex PCR, libraries were generated by adapter ligation and target enrichment using the Gene Read DNA Library I Core Kit, the Gene Read DNA I Amp Kit (Qiagen), and the NEXTflex DNA Barcodes (Bio Scientific). 12 pM of the constructed libraries were sequenced on the MiSeq platform (Illumina) with a MiSeq reagent kit V2 (Illumina) with 300 cycles following the manufacturer’s recommendations. Data analysis and mutation calling were performed as previously described [32]. The following genes and gene regions were evaluated for mutations: *ALK* (Exons 21–25), *AKT1* (Exon 4), *BRAF* (Exons 11 and 15), *CTNNB1* (Exon 3), *DDR2* (Exons 3–18), *EGFR* (Exons 18–21), *HER2* (Exons 19 and 20), *KRAS* (Exons 2 and 3), *MEK1* (*MAP2K1*) (Exon 2), *MET* (Introns 13/14, Exon 14), *NRAS* (Exons 2 and 3), *PIK3CA* (Exons 9 and 20), *PTEN* (Exons 1–9), *TP53* (Exons 5–8).

A subset of samples was further analyzed with the QIAseq targeted DNA panel for human lung cancer (NGHS-005X-96) with the GeneRead DNAseq Panel PCR Kit V2 (Qiagen). Libraries were prepared using the Gene Read DNA Library I Core Kit and the Gene Read DNA I Amp Kit (Qiagen) according to manufacturer’s protocol. Barcoded libraries were amplified, final library products were quantified, diluted, and pooled in equal amounts. Finally, 1, 2 pM of the final libraries were sequenced on a NextSeq Sequencer (Illumina, San Diego, CA, USA) with the NextSeq 500 Mid Output Kit v2 following manufacturer’s recommendations. The following regions were analyzed: *ALK* (Exons 2–15, 17–29), *ATM* (Exons 2–13, 15–45, 47–63), *BRAF* (Exons 2–4, 6–18), *EGF*R (Exons 2–26, 28), *ERBB2* (Exons 1, 3, 5–6, 8–12, 14–26), *ERBB4* (Exons 1–6, 8–13, 15–28), *FGFR1* (Exons 2, 4–10, 12–18), *FGFR2* (Exon 2–18), *KDR* (Exon 2–20, 22–30), *KEAP1* (Exon 3–6), *KIT* (Exon 2–21), *KRAS* (Exons 2–6), *MET* (Exons 2–21), *NFE2L2* (Exons 2–5), *PDGFRA* (Exons 2–23), *PIK3CA* (Exons 2–10, 12–16, 18–21), *PIK3CG* (Exons 3–10), *RET* (Exons 2–10, 12–20), *ROS1* (Exons 1–43), *SMARCA4* (Exons 2–5, 7–17, 19–27, 29, 30, 32–35), *STK11* (Exons 1–2, 4–8), *TP53* (Exons 2, 4–11).

The generated libraries were equimolarly pooled for amplicon sequencing to a concentration of 3 nM of each sample to counterbalance differences in sample quality. Sequencing was performed on an Illumina MiSeq benchtop sequencer (Illumina, San Diego, USA). Results were visualized in the Integrative Genomics Viewer (IGV) and manually analyzed with an in-house pipeline. Results were only interpreted if the coverage was >200.

### Digital droplet PCR

In a digital droplet PCR (ddPCR), the reaction is split into ~12–15,000 individual droplets, each containing zero or one or more DNA molecules. During the readout, each droplet is individually counted and accessed for fluorescence. In each experiment, a DNA sample with the confirmed *ALK* mutation served as a positive control. The number of wildtype DNA molecules was determined in the same reaction using a second probe complementary to the wildtype sequence of the tested gene. Amplifications were carried out in duplicates and a reaction volume of 20 μL on the QX200 Droplet Digital PCR System (Bio-Rad). Each PCR reaction contained 3 μL PCR grade water, 10 μL Bio-Rad PCR mix for probes, 1 μL of each (target and reference) amplification primer/probe mix, and 10 ng of genomic DNA. PCR cycling was performed on a C1000 thermo cycler (Bio-Rad) according to manufacturer’s instructions. A minimum of 200 wildtype droplets was necessary for analyses. Results were analyzed with Quantasoft v.1.3.2 software (Bio-Rad) and reported as allelic fractions.

### Statistics

Statistical analyses were performed using IBM SPSS (version 27) and R (version 4.1.2) [33]. Fisher’s exact test (two-sided) was used to assess occurrence of *ALK* alterations in different subgroups if the test was performed between two groups, or Chi-squared test for comparisons between more than two groups. Median, first and third quartile of age at diagnosis in different subgroups are presented as boxplots with whiskers displaying the minimum and maximum of the data within 1.5 times the IQR (interquartile range). A Wilcoxon-Mann-Whitney (two-sided) test was used to compare median ages. Kaplan-Meier curves showing survival estimates were compared using the log-rank test in case the proportional-hazards assumption hold and Gehan-Breslow (generalized Wilcoxon) test otherwise. Additionally, probabilities of 5-year event-free survival (EFS) and overall survival (OS) along with their 95% confidence intervals basing on the cumulative hazard were extracted from the Kaplan-Meier estimates. Results of statistical tests with *P* values below 0.05 are regarded as being significant.

## Results

To comprehensively determine the spectrum of *ALK* alterations in neuroblastomas at diagnosis and at relapse, we retrospectively collected data on *ALK* mutations and/or amplifications from tumors of 943 neuroblastoma patients. Longitudinal samples from the same patient were available for subcohorts. Patients were treated in successive GPOH trials and covered the entire spectrum of the disease, including high-risk and non-high-risk patients, as well as all stages of the International Neuroblastoma Staging System (INSS; Fig. 1, Supplementary Table 1).

### ALK alterations in neuroblastoma at diagnosis

**Fig. 2:**
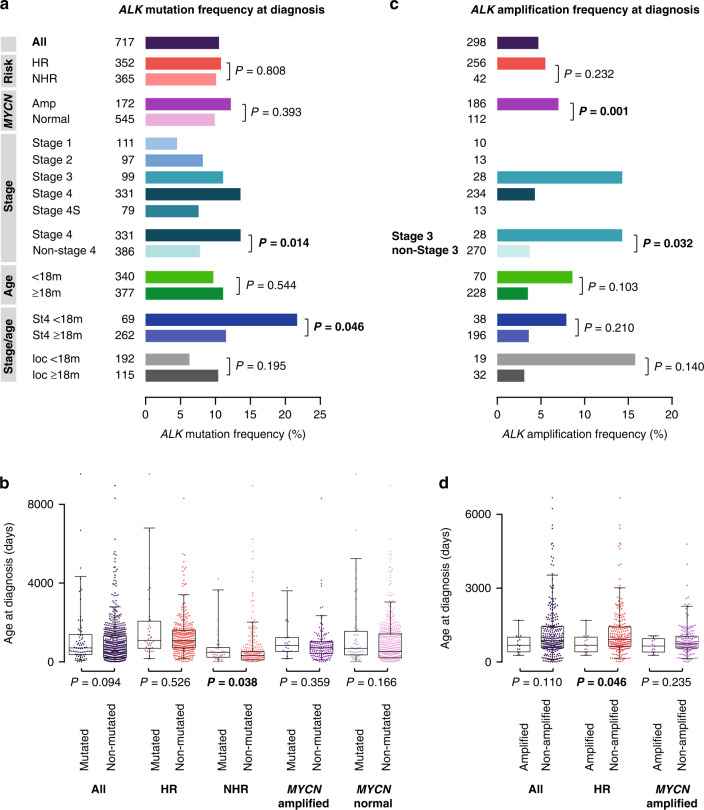
*ALK* alterations in neuroblastoma at diagnosis. **a** *ALK* mutation frequencies in neuroblastoma obtained at diagnosis. **b** Age at diagnosis of patients with *ALK*-mutated *versus* non-mutated neuroblastoma obtained at diagnosis. **c** *ALK* amplification frequencies in neuroblastoma obtained at diagnosis. **d** Age at diagnosis of patients with *ALK*-amplified *versus* non-amplified neuroblastoma obtained at diagnosis. Boxplots show the median, first and third quartile (boxes), with whiskers indicating the minimum and maximum of the data within 1.5× the interquartile range. *P* values for **a** and **c** were calculated using Fisher’s exact test or Chi-squared test where appropriate, and for **b** and **d** using Wilcoxon–Mann–Whitney test. HR, high-risk; NHR, non-high-risk; ampl, amplified; m, months; St4, stage 4; loc, localized disease.

In neuroblastomas obtained at diagnosis, we detected SNV in the *ALK* kinase domain in 10.5% (75/717) of the cases (Fig. 2a). These mutations were equally distributed across the high-risk and non-high-risk groups (10.8% versus 10.1%, *P* = 0.808), and mutation frequencies did also not differ in *MYCN*-amplified and non-amplified tumors (12.2% *versus* 9.9%, *P* = 0.393). *ALK* mutations were significantly enriched in stage 4 tumors compared to non-stage 4 tumors (13.6% versus 7.8%, *P* = 0.014), with lowest frequencies occurring in stage 1 (4.5%) and stage 4S (7.6%) tumors (Fig. 2a). *ALK* mutation frequencies did not differ in patients <18 months and ≥18 months of age (9.7% versus 11.1%, *P* = 0.544, Fig. 2a). We noted, however, that *ALK* mutations occurred significantly more frequently in stage 4 tumors of patients <18 months than of patients ≥18 months (21.7% *versu*s 11.5%, *P* = 0.046, Fig. 2a), with highest frequencies in *MYCN* non-amplified tumors of the former subgroup (9/35 cases, 25.7%; Supplementary Table 1). In general, age was not associated with *ALK* mutation status in the entire cohort (Fig. 2b). By contrast, patients of the non-high-risk cohort were significantly older when having *ALK*-mutated tumors in comparison to *ALK* wildtype tumors (15.8 versus 10.6 months, *P* = 0.038, Fig. 2b).

*ALK* amplification was detected in 4.7% of neuroblastomas obtained at diagnosis (14/298, Fig. 2c). However, this cohort was biased towards high-risk patients and may thus overestimate the overall frequency of such alterations in neuroblastoma. In the high-risk cohort and in *MYCN*-amplified tumors, *ALK* amplifications occurred in 5.5% (14/256) and 7.6% (14/185), respectively. *ALK* amplifications were not detected in non-high-risk tumors, nor in tumors without *MYCN* amplification. *ALK* amplifications occurred only in stage 3 (4/28, 14.3%) and stage 4 (10/234, 4.3%) neuroblastomas, and were significantly enriched in stage 3 compared to non-stage 3 tumors (14.3% versus 3.7%, *P* = 0.032, Fig. 2c). Age at diagnosis of patients having *ALK*-amplified tumors did not differ from that of patients with non-amplified tumors in the entire cohort (22.2 versus 28.2 months, *P* = 0.110, Fig. 2d), however, patients with high-risk disease showing *ALK* amplification were significantly younger than high-risk patients with tumors lacking *ALK* amplification (22.2 versus 29.9 months, *P* = 0.046).

### ALK alterations in neuroblastoma at relapse

**Fig. 3:**
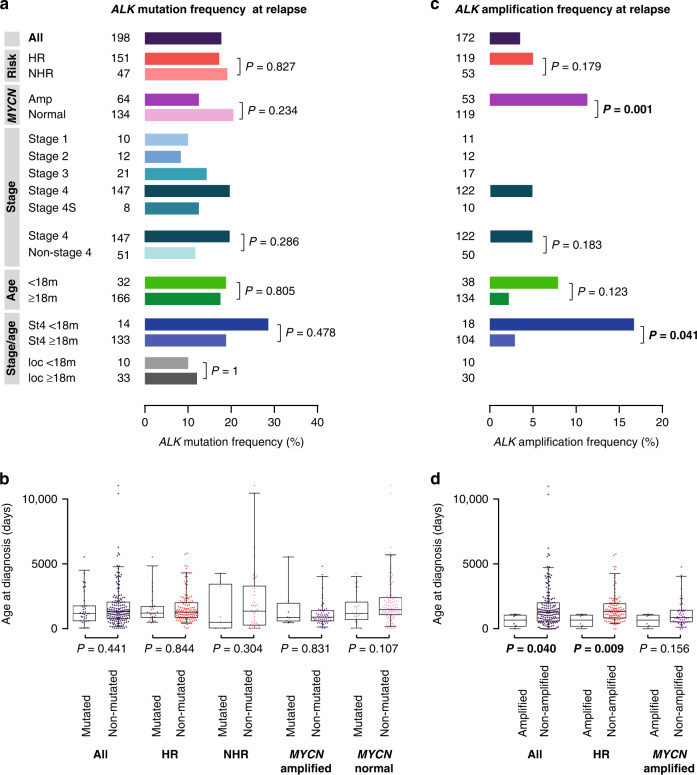
*ALK* alterations in neuroblastoma at relapse. **a** *ALK* mutation frequencies in relapsed neuroblastoma. **b** Age at diagnosis of patients with *ALK*-mutated versus non-mutated relapsed neuroblastoma. **c** *ALK* amplification frequencies in relapsed neuroblastoma. **d** Age at diagnosis of patients with *ALK*-amplified versus non-amplified relapsed neuroblastoma. Boxplots show the median, first, and third quartile (boxes), with whiskers indicating the minimum and maximum of the data within 1.5× the interquartile range. *P* values for **a** and **c** were calculated using Fisher’s exact test or Chi-squared test where appropriate, and for **b** and **d** using Wilcoxon–Mann–Whitney test. HR, high-risk; NHR, non-high-risk; ampl, amplified; m, months; St4, stage 4; loc, localized disease.

To assess *ALK* alterations in neuroblastoma at relapse or progression, we investigated *ALK* SNV and amplification in 198 and 172 tumors, respectively (Fig. 1). We found *ALK* SNV in 17.7% of the cases (35/198, Fig. 3a). The *ALK* mutation frequency at relapse did not differ between high-risk and non-high-risk patients (17.2% versus 19.1%, *P* = 0.827). We also did not observe differences in *ALK* mutation frequencies at relapse between *MYCN*-amplified and *MYCN* non-amplified tumors (12.5% versus 20.1%, *P* = 0.234), between tumors at stage 4 and other stages (19.7% versus 11.8%, *P* = 0.286), and between tumors from patients of different ages (Fig. 3a, b).

*ALK* amplification was detected in 3.5% (6/172) of all relapsed tumors (Fig. 3c). In the high-risk subgroup, 5.0% (6/119) harbored amplification of *ALK*, all of which occurred in *MYCN-*amplified tumors (6/53) and were thus significantly enriched in this subgroup (11.3% versus 0%, *P* = 0.001). All patients with tumors showing *ALK* amplification at relapse had stage 4 disease (6/122 patients with stage 4 disease, 4.9%). Patients with tumors bearing *ALK* amplification at relapse were significantly younger than patients with *ALK* non-amplified tumors in the entire cohort (23.0 versus 42.7 months, *P* = 0.040, Fig. 3d) and in the subgroup of high-risk patients (23.0 versus 44.6 months, *P* = 0.009), while it did not differ between *ALK*-amplified and non-amplified tumors in the subgroup of patients with *MYCN*-amplified neuroblastoma (23.0 versus 29.0 months, *P* = 0.156). We noted, however, that *ALK* amplification frequencies at relapse were significantly elevated in patients <18 months with stage 4 disease as compared to patients ≥18 months with stage 4 neuroblastoma (16.7% versus 2.9%, *P* = 0.041, Fig. 3c).

### Differences of ALK alteration frequencies in neuroblastoma at diagnosis and relapse

**Fig. 4:**
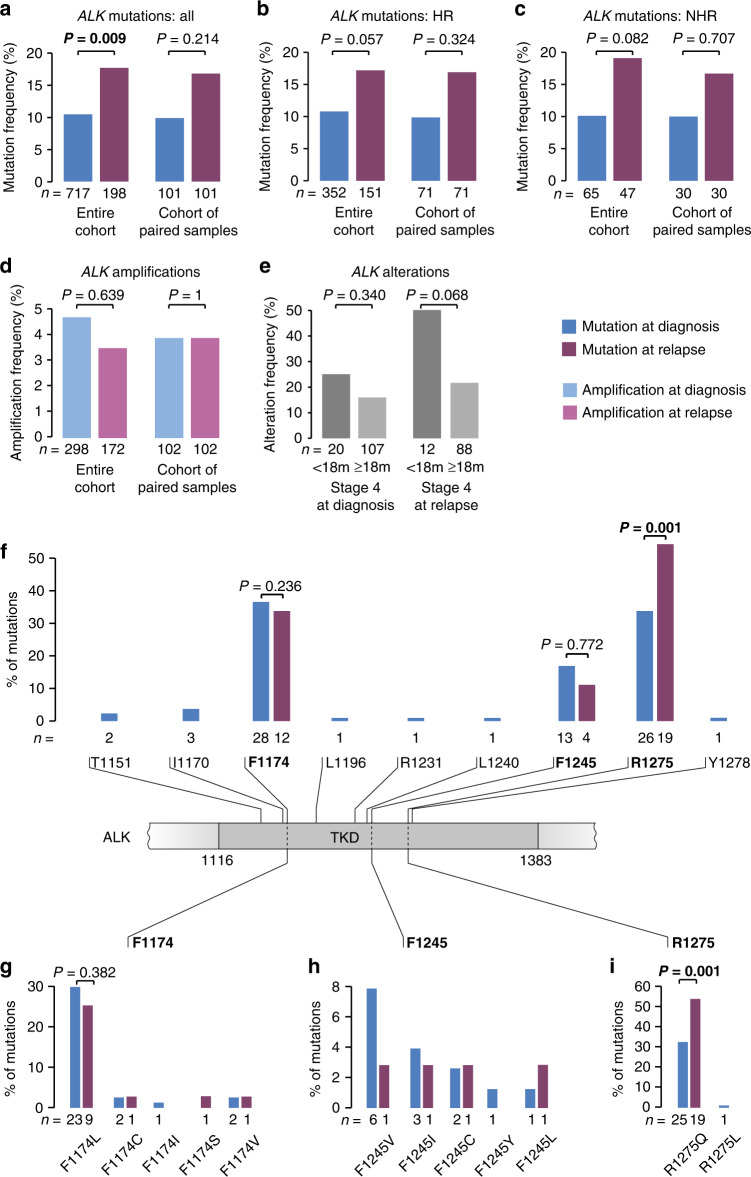
Comparison of *ALK* alteration frequencies in neuroblastoma obtained at diagnosis and at relapse. **a** *ALK* mutation frequencies in neuroblastoma obtained at diagnosis versus relapse in the entire cohort (left) and in the subgroup of patients of whom paired samples were available (right). **b** *ALK* mutation frequencies in neuroblastoma obtained at diagnosis versus relapse in high-risk tumors (HR) in the entire cohort (left) and in the subgroup of patients of whom paired samples were available (right). **c** *ALK* mutation frequencies in neuroblastoma obtained at diagnosis versus relapse in non-high-risk tumors (NHR) in the entire cohort (left) and in the subgroup of patients of whom paired samples were available (right). **d** *ALK* amplification frequencies in neuroblastoma obtained at diagnosis versus relapse in the entire cohort (left) and in the subgroup of patients of whom paired samples were available (right). **e** *ALK* alteration frequencies in stage 4 neuroblastoma of patients <18 months versus ≥18 months at diagnosis in the cohort of tumors obtained at diagnosis (left) and at relapse (right). **f** Schematic representation of the ALK tyrosine kinase domain (TKD) and the amino acid positions and frequencies of mutations detected in neuroblastoma samples at diagnosis and at relapse. **g** Frequencies of mutation types at position F1174 at diagnosis and at relapse. **h** Frequencies of mutation types at position F1245 at diagnosis and at relapse. **i** Frequencies of mutation types at position R1275 at diagnosis and at relapse. Of note, mutation type of one mutation detected in a tumor at relapse had remained unknown. *P* values were calculated using Fisher’s exact test.

Evaluation of changes in *ALK* mutation and amplification frequencies over the course of disease revealed a significant increase of *ALK* SNV at relapse in the entire cohort (10.5% versus 17.7%, *P* = 0.009, Fig. 4a). A similar increase in potential de novo mutations was observed in the subgroup of paired samples (*n* = 101; 9.9% versus 16.8%, *P* = 0.214). Both tumors of high-risk (10.8% versus 17.2%, *P* = 0.057, Fig. 4b) and non-high-risk patients (10.1% versus 19.1%, *P* = 0.082, Fig. 4c), comprising both low- and intermediate-risk cases, were affected by the increment of mutations over time, at almost identical rates.

*ALK* amplification frequencies did neither differ between tumors at diagnosis and relapse in the entire cohort (4.7% versus 3.5%, *P* = 0.639; Fig. 4d), nor in the subgroup of *MYCN*-amplified tumors (7.0% versus 11.3%, *P* = 0.386). In line with that finding, no de novo amplifications were found in the subgroup of paired samples obtained from 102 patients (*ALK* amplification in 4/102 cases, 3.9%). Information on both *ALK* mutation and amplification status was available for 310 samples (167 at diagnosis, 143 at relapse), 58 of which showed any type of *ALK* alteration (18.7%). In this cohort, we observed a particular high frequency of *ALK* alterations in tumors of patients with stage 4 disease <18 months of age, with 25% (5/20) of tumors being affected at diagnosis and 50% (6/12) at relapse (Fig. 4e). *ALK* mutations and amplifications occurred mutually exclusive in all samples investigated.

Allelic fractions were available for 62/76 and 24/36 *ALK* mutations detected at diagnosis and relapse, respectively, as some samples had been analyzed by dideoxy-sequencing only (Supplementary Table 1). At diagnosis, 2/62 mutations (3.2%) were detected at allelic fractions below 5%, and 15 (24.2%) at allelic fractions ≤20% (Supplementary Fig. 1a). At relapse, all mutations were detected at allelic fractions >5%, and 6/24 (25.0%) were found at allelic fractions ≤20% (Supplementary Fig. 1b). Allelic fractions (>20% versus ≤ 20%) were not associated with prognostic variables, such as *MYCN* amplification status (*MYCN*-amplified versus non-amplified, *P* = 0.332), risk group (high-risk *versus* non-high-risk, *P* = 1), stage (stage 4 versus stage 1–3/4S, *P* = 0.362) and age (≥18 versus < 18 months, *P* = 0.235, Supplementary Fig. 1c). Allelic fractions did also not differ between patients with stage 4 disease <18 months versus ≥ 18 months (*P* = 0.443, Supplementary Fig. 1d).

The vast majority (88.2%, 67/76) of *ALK* mutations at diagnosis and all mutations at relapse (100%, 36/36) were detected at the three common hotspot positions within the tyrosine kinase domain, i.e., R1275, F1174, and F1245 (Fig. 4f). In tumors at diagnosis, mutations also rarely affected other positions within the kinase domain, i.e., T1151, I1170, L1196, R1231, L1240, and Y1278 (Fig. 4f). F1174 was the position most frequently affected by mutations in tumors at diagnosis (28/76), showing an exchange of phenylalanine to leucine in most cases (Fig. 4g). The majority of mutations at position F1245 led to an exchange of phenylalanine to valine (6/13, Fig. 4h). Mutations at R1275 resulted in arginine to glutamine substitutions in all but one case and were the most frequent SNV in relapsed tumors (19/36, Fig. 4i). Of note, the frequency of R1275Q mutations increased significantly at relapse (32.9% versus 52.8%, *P* = 0.001, Fig. 4i).

### Impact of ALK alterations on neuroblastoma patient outcome

**Fig. 5:**
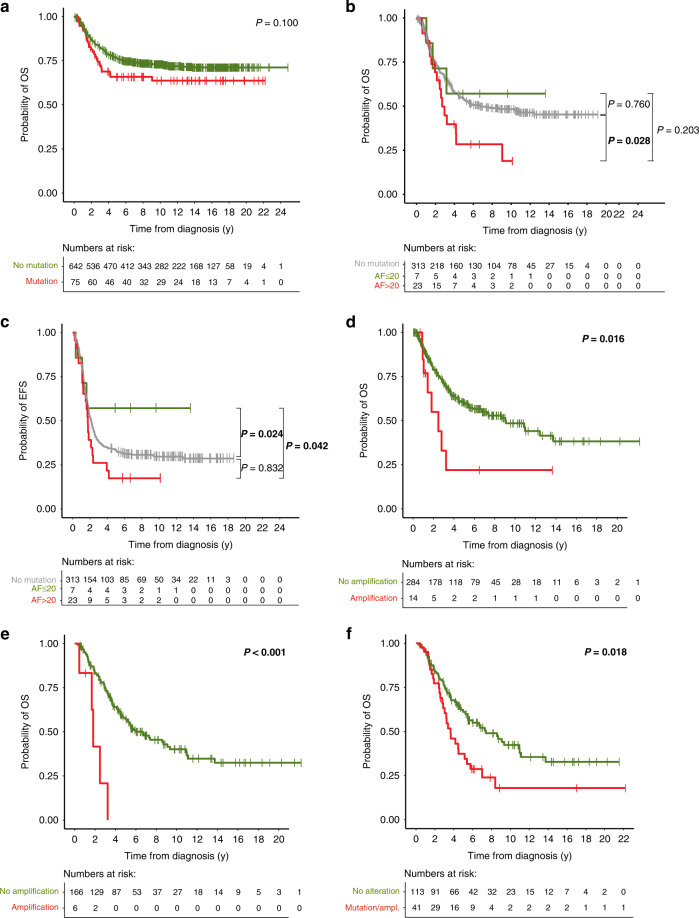
Impact of *ALK* alterations on patient survival. **a** OS of patients with *ALK*-mutated tumors versus patients with *ALK* wildtype tumors at diagnosis (5-year OS, 66% versus 76%). **b** OS of high-risk patients with tumors harboring *ALK* mutations at allelic fractions >20% versus ≤ 20% versus ALK wildtype (5-year OS, 28% versus 57% versus 54%). **c** EFS of high-risk patients with tumors harboring *ALK* mutations at allelic fractions >20% versus ≤ 20% versus ALK wildtype (5-year EFS, 17% versus 57% versus 33%). **d** OS of patients with *ALK*-amplified tumors versus ALK non-amplified tumors at diagnosis (5-year OS, 22% versus 60%). **e** OS of patients with *ALK*-amplified versus non-amplified tumors at relapse (5-year OS, 0% versus 57%). **f** OS of patients with *ALK*-altered versus ALK-non-altered tumors at relapse (5-year OS, 37% versus 61%). *P* values were calculated by log-rank and, in case of non-proportional hazards, Gehan-Breslow test. OS, overall survival; y, years; ampl, amplification; AF, allelic fraction.

The presence of *ALK* SNV in tumors obtained at diagnosis was not prognostic for both OS and EFS in the entire cohort (Fig. 5a, Supplementary Fig. 2a), the non-high-risk subgroup (Supplementary Fig. 2b, c), and the high-risk subgroup (Supplementary Fig. 2d, e). Strikingly high *ALK* mutation frequencies were found in tumors of patients with stage 4 disease younger 18 months of age, however, survival of these patients did not differ from that of corresponding patients lacking *ALK* mutations (Supplementary Fig. 3a, b). We also found no difference in outcome of patients with tumors bearing *ALK* mutations at allelic fractions >20% versus ≤ 20% *versus* no mutation in the entire cohort (Supplementary Fig. 4a, b). In high-risk patients, however, OS of patients with tumors harboring *ALK* mutations at allelic fractions >20% was significantly worse than that of patients whose tumors lacked an *ALK* mutation (Fig. 5b). EFS of high-risk patients with tumors harboring *ALK* mutations at allelic fraction ≤20% was significantly better than that of patients with tumors harboring *ALK* mutations at allelic fractions >20% or without *ALK* mutations (Fig. 5c). OS of patients with *ALK*-amplified tumors obtained at diagnosis was significantly poorer than that of patients with *ALK* non-amplified tumors (Fig. 5d), with a similar trend in the high-risk subgroup (Supplementary Fig. 5a), whereas *ALK* amplification was not prognostic for EFS in the entire cohort and in the high-risk subgroup (Supplementary Fig. 5b, c). As *ALK* amplification exclusively occurred in *MYCN*-amplified tumors, we also examined patient survival in this subgroup. We found that patients with *ALK*-amplified tumors appear to have poorer OS and EFS than those without *ALK* amplification, however, the differences did not reach significance (Supplementary Fig. 5d, e). We also assessed the prognostic relevance of *ALK* alterations, i.e., both mutations and amplification, within the subgroup of high-risk patients, however, we did not find significant impact on OS and EFS (Supplementary Fig. 6a, b).

In patients with relapsed tumors, *ALK* mutations did not significantly affect OS or EFS in the entire cohort (Supplementary Fig. 7a, b), the non-high-risk subgroup (Supplementary Fig. 7c, d), and the high-risk subgroup (Supplementary Fig. 7e, f). The presence of *ALK* amplification at relapse was associated with fatal outcome, since all such patients had died. In detail, patients with *ALK*-amplified tumors at relapse had significantly poorer OS (Fig. 5e) and EFS in the entire cohort (Supplementary Fig. 8a), as well as in the high-risk subgroup (Supplementary Fig. 8b, c). In the subgroup of patients with *MYCN*-amplified tumors, *ALK* amplification at relapse was significantly associated with poorer EFS (Supplementary Fig. 8d), but not OS (Supplementary Fig. 8e). We found that genomic *ALK* alterations (i.e., both mutations and amplification) at relapse were associated with impaired OS and EFS in the entire group (Fig. 5f and Supplementary Fig. 9a, respectively), and with impaired EFS, but not OS, in the subgroup of high-risk patients (Supplementary Fig. 9b, c, respectively). *ALK* alterations at relapse were also associated with poorer secondary OS, calculated from the timepoint of relapse, in the entire cohort (Supplementary Fig. 10a), but not in the subgroup of high-risk patients (Supplementary Fig. 10b).

Outcome of neuroblastoma patients did not significantly depend on the type of *ALK* mutations, although OS of patients with F1174L mutations tended to be slightly worse than that of patients with tumors harboring other *ALK* mutations or *ALK* wildtype tumors (Supplementary Fig. 11a). We did not observe any associations of specific *ALK* mutations with *MYCN* amplification in tumors obtained at diagnosis (Supplementary Fig. 11b, c). In relapsed tumors, however, R1275Q mutations were predominantly found in *MYCN* non-amplified tumors (Supplementary Fig. 11b), and F1174L mutations tended to be associated with *MYCN* amplification (Supplementary Fig. 11c).

### Dynamics of ALK alterations in neuroblastoma over the course of disease

**Fig. 6:**
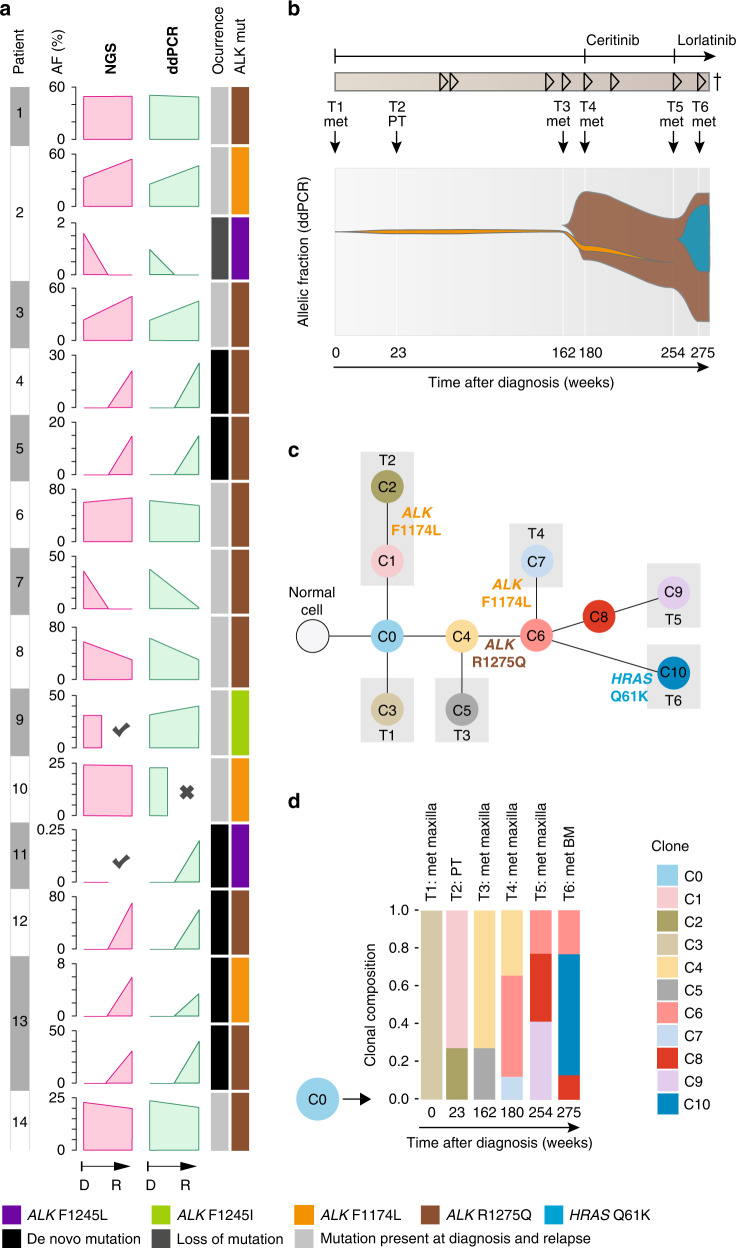
Dynamics of *ALK* alterations over the course of disease. **a** Comparison of allelic fractions of *ALK* mutations detected at diagnosis and relapse by NGS versus ddPCR. In two patients (P2 and P13), two different *ALK* mutations were detected in the same tumor samples. X indicates that no material was available, check marks indicate that mutations were detected by dideoxy-sequencing only, thus leaving their allelic fraction unknown. **b** Longitudinal monitoring of allelic fractions (as determined by ddPCR) of two different *ALK* mutations (R1275Q and F1174L) over the course of disease in an individual patient. The allelic fraction of the *HRAS* mutation occurring at week 275 was determined by panel NGS. ALK inhibitory treatment and the clinical course of disease are shown at the top, with arrowheads indicating progression or relapse of disease. Timepoints and localizations of biopsies are indicated by arrows; biopsies of metastasis were taken from the same maxillary metastasis, except the last biopsy, which was derived from bone marrow. **c** Schematic diagram for the clonal evolution of cancer cell populations reconstructed from whole-exome sequencing data in patient P13. T1–T6 represent the consecutive biopsies that were taken from the patient. Gray boxes highlight clones that were private to the respective biopsy. *ALK* and *HRAS* mutations are indicated as they appeared in chronological order. **d** Clonal composition of each biopsy. The ancestral clone C0 is illustrated on the left to indicate its presence in every biopsy. AF, allelic fraction; NGS, next generation sequencing; ddPCR, digital droplet PCR; mut, mutation; D, diagnosis; R, relapse; met, metastasis; PT, primary tumor; BM, bone marrow.

To investigate the dynamics and clonal evolution of *ALK* aberrations over the course of disease, we determined *ALK* alterations in longitudinal samples, taken at diagnosis and at relapse or progression. *ALK* mutations were analyzed in 202 samples (101 diagnostic/relapse pairs) by panel NGS (*n* = 71), dideoxy-sequencing (*n* = 27), WES (*n* = 44), or a combination of at least two of these methods (*n* = 60). As outlined above, we found *ALK* SNV in 10/101 tumors (9.9%) at diagnosis, and in additional 7 tumors (total, 17/101 cases, 16.8%) at relapse (Fig. 4a). To further assess clonal development of mutations detected within this cohort, allelic fractions of mutations were determined by mutation-specific ddPCR. We found that allelic fractions detected by ddPCR matched well with allelic fractions determined by massively parallel sequencing (Fig. 6a). In addition, this approach validated all mutations identified by dideoxy-sequencing or panel NGS, except in one sample (patient P7). In this case, an R1275Q mutation detected at diagnosis was not detected by panel NGS but by ddPCR at relapse at an allelic fraction of 0.35% (Fig. 6a), which might be due to low tumor infiltration in the relapse sample (5%). In two cases, we found two distinct *ALK* mutations within the same tumor. One tumor harbored an F1174L mutation at diagnosis and at relapse, and an additional F1245L mutation at low allelic fraction at diagnosis, which had disappeared at relapse (patient P2, Fig. 6a). In another tumor, two different ALK de novo mutations occurred during the course of the disease (R1275Q and F1174L, patient P13, Fig. 6a, see below). Potential de novo ALK mutations at relapse were mostly R1275Q mutations (*n* = 4), while F1174L, F1174C, F1245L, and F1245C mutations were detected in one sample each. Allelic fractions of R1275Q mutations were higher than those of other de novo mutations, however, the number of cases was too small to draw final conclusions (Supplementary Fig. 11d).

Patient P13 was particularly informative for analyzing the clonal evolution of *ALK* mutations in neuroblastoma, as tumor material of six different timepoints was available for monitoring allelic fractions over the course of disease by ddPCR (Fig. 6b). While *ALK* was wild-type in the metastatic tumor at diagnosis, an F1174L mutation occurred at low allelic fractions in the second biopsy which was derived from the primary tumor. This mutation was also found in the fourth biopsy, obtained from a maxillary metastasis, but not at any other timepoint. In addition, an R1275Q mutation occurred in the fourth biopsy with an allelic fraction of 40.5%, which gradually increased to nearly 80% in the sixth biopsy. The patient received treatment with the ALK inhibitor ceritinib [4] after the fourth biopsy, which was changed to lorlatinib after the fifth biopsy. In the sixth biopsy, the tumor had acquired an additional *HRAS* mutation at codon 61 (Q61K), and the patient died shortly afterwards due to disease progression [34]. Reconstruction of the clonal evolution of this tumor revealed that all *ALK* mutations and the *HRAS* alteration had developed from the same ancestral clone C0 (Fig. 6c). Surprisingly, the two clones C2 and C7 that harbored subclonal F1174L mutations did not share common ancestors besides C0, indicating that the two F1174L mutations had developed independently. By contrast, the last three timepoints were dominated by clones that carried the R1275Q mutation, with C6 representing the ancestor of all subsequent clones (Fig. 6c, d). Together, the chronology of the occurrence of mutations in this tumor indicates that *ALK* F1174L mutations may not necessarily prevail in neuroblastoma, both in the absence and presence of a concurrent *ALK* R1275Q mutation.

## Discussion

We here provide a detailed view on the development of genomic *ALK* alterations in neuroblastoma over the course of the disease. We found an overall *ALK* mutation frequency of 10.5% in a large and representative cohort of neuroblastomas obtained at diagnosis, which is largely in the range of *ALK* mutation frequencies reported previously [8–15]. In our study, the prevalence of *ALK* mutations did not differ between clinical risk groups, which has been controversially discussed in earlier studies, some of which had suggested that *ALK* mutations may be more prevalent in high-risk group tumors [5, 12, 18, 19, 35]. In addition, we did not find enrichment of *ALK* mutations in *MYCN*-amplified neuroblastomas [15, 19]. We noticed, however, that *ALK* mutation frequencies were highest in stage 4 tumors, and in stage 4 tumors of patients younger than 18 months of age in particular. The enrichment of *ALK* mutations in the latter subgroup, which is considered as intermediate risk if *MYCN* amplification is absent, has not been reported in previous studies [11, 12, 18, 19, 36–38]. *ALK* mutations were not prognostic in our study, except in high-risk patients when allelic fractions were taken into account. In the latter analysis, *ALK* mutations were associated with poorer outcome when they occurred at allelic fractions >20%, which is in line with previous results [15].

*ALK* amplification was found in 4.7% of all tumors and 5.5% of high-risk tumors at diagnosis, which is in line with the frequency of *ALK* amplifications reported previously [5, 11, 12, 15, 17–19]. Similar to other studies, *ALK* amplification occurred exclusively in *MYCN*-amplified neuroblastomas and were mutually exclusive with *ALK* point mutations [5, 19, 39]. In addition, we noted that *ALK* amplification was associated with young patient age and with stage 3 in tumors obtained at diagnosis. Patients whose tumors harbored *ALK* amplification had worse overall survival, supporting the notion that these tumors constitute a highly aggressive subtype of neuroblastoma [12].

Only few studies with limited patient numbers have reported on *ALK* mutation and amplification frequencies in relapsed or progressive neuroblastoma [10, 40, 41], despite their potential relevance as therapeutic targets for these patients with poor survival expectations. In a cohort of roughly 200 cases, we found *ALK* point mutations in 17.7% of tumors at relapse or progression, which was a significant increase compared to cases at diagnosis. Of note, the mutation frequency of the entire cohort closely matched the frequency detected in relapsed tumors of the sub-cohort of patients for whom paired samples from diagnosis and relapse were available (*n* = 101), suggesting that *ALK* mutations may occur de novo in 7% of relapsed or progressive neuroblastoma. We also found that mutation frequencies increased at similar rates in high-risk and non-high-risk tumors, indicating that *ALK* mutations may develop under various conditions of selective pressure, ranging from limited to high treatment intensities. In a previous study of 54 paired neuroblastoma samples, an increase of the *ALK* mutation frequency from 16.7% at diagnosis to 25.9% at relapse has been reported [10]. A deep genome sequencing approach in this study revealed de novo ALK mutations in only 3.7% at relapse, whereas some *ALK* mutations were found at sub-clonal levels already at diagnosis. This finding is in contrast to our observations, as we could not detect putative de novo mutations of relapse samples in their corresponding diagnostic counterparts with highly sensitive mutation-specific ddPCR assays. It has to be noted, though, that we cannot formally exclude that mutations had been present already at the time of diagnosis in very minor subclones or in tumor regions other than the biopsy sample. Another study of 23 paired samples revealed *ALK* mutations in 30.4% at diagnosis and 43.5% at relapse, however, the small patient number limits its informative value on *ALK* mutation frequencies in relapsed neuroblastoma, and no attempts to assess sub-clonality had been made [40].

We found that the type of mutation occurring most frequently in relapsed neuroblastomas was R1275Q, with a significant increase of such mutations compared to tumors at diagnosis. The abundance of these mutations at relapse was probably caused by de novo occurrence during the course of disease, as we detected R1275Q mutations in four of the seven tumors that harbored *ALK* mutations only at relapse. In addition, we did not find clear associations of mutation types with patient outcome, arguing against significant enrichment effects of R1275Q mutations through disproportionally high relapse rates in tumors bearing such mutations. We also noted that R1275Q mutations at relapse were more prevalent in *MYCN* non-amplified tumors, while F1174L tended to be associated with *MYCN-*amplified disease, as described previously [9, 11, 15]. Detailed analyses of clonal evolution of two *ALK* mutations that emerged in an individual tumor revealed F1174L mutations at low allelic fractions in the second and fourth biopsy, and an R1275Q mutation at high allelic fractions from the time of fourth biopsy to the time of final progression. These data demonstrate that (i) the presence of an *ALK* mutation may not always provide selective advantage in neuroblastoma, and (ii) the mutation with the putatively highest oncogenic potential F1174L [11] does not necessarily prevail over the R1275Q mutation, both under conventional treatment and ALK inhibitor therapy.

Treatment strongly affects neuroblastoma patient outcome and may therefore be a confounding factor in prognostic biomarker studies. The fact that patients from successive GPOH trials or registries with evolving treatment strategies had been analyzed in this study may thus have biased the results. It has to be noted, though, that the vast majority of patients had been treated within the trial NB2004 and the subsequent registry NB2016, which kept the therapeutic concept of NB2004, resulting in homogeneous treatment of most patients included in this study. Heterogeneity of diagnostic procedures used for detection of *ALK* mutations may be another limiting factor of this study. A fraction of cases had been analyzed by dideoxy-sequencing only, which has a lower sensitivity than NGS-based approaches [8, 9]. Sequencing results from 125 samples examined by both dideoxy-sequencing and approaches with higher sensitivity (i.e., NGS or ddPCR), however, were concordant in 99.2% (124/125), with dideoxy-sequencing detecting *ALK* mutations at surprisingly low allelic fractions going below 5% (Patient P652, Supplementary Table 1). In fact, we found only one mutation by ddPCR at an allelic fraction of 2.3% that had been missed by dideoxy-sequencing, suggesting that mutation frequencies reported in this study are accurate. Nevertheless, we would prefer NGS-based methods for *ALK* diagnostics in future studies, as they provide comprehensive genetic information with high sensitivity and precise allelic fractions of mutations in a single approach. Other factors that may have affected the detection sensitivity of *ALK* alterations are low tumor cell contents in some samples and spatial heterogeneity of genetic alterations within the tumor. Finally, small sample sizes in specific subgroups, such as the cohorts of *ALK* amplified tumors or young patients with stage 4 disease, may limit interpretation of the study results.

Considering the high incidence of putative de novo ALK point mutations at relapse and their potential therapeutic consequences, we suggest to determine the genomic *ALK* status in all neuroblastoma cases at relapse or progression. Extensive testing might be even more important as we found that R1275Q mutations were most frequently detected at relapse, a mutation that has been reported to be sensitive to ALK inhibitory treatment [4, 42]. By contrast, assessment of *ALK* amplification in relapsed disease may be dispensable if already determined at diagnosis, as novel *ALK* amplification was not detected at relapse. In addition, we suggest to also assess genomic *ALK* alterations systematically in intermediate-risk patients at diagnosis, which have been underappreciated in previous studies. The finding that intermediate-risk neuroblastomas of young stage 4 patients at diagnosis harbor *ALK* mutations at high frequencies provides a starting point for targeted treatment of these children. While these patients have overall favorable outcome with current therapies [43, 44], it has to be noted that they receive quite intensive cytotoxic treatment, which is associated with substantial toxicities and long-term sequelae. We therefore suggest to evaluate the therapeutic benefit of ALK inhibitors in intermediate-risk patients with *ALK*-mutated neuroblastoma in prospective clinical trials.

## Supplementary information

Supplementary Material

Supplementary Table 1

Supplementary Table 2

## Data Availability

Patient whole-genome and whole-exome sequencing data have been published previously and are deposited at the European Genome-phenome Archive (https://ega-archive.org) under study accession numbers EGAS00001001308 and EGAS00001003244, respectively. Sequencing data of INFORM patients is available via accession number EGAS00001005112. Due to the sensitive nature of these patient datasets, the WGS data is subject to approval by the data provider. Please see the corresponding EGA data access committee (DAC) for more details on the procedure (https://ega-archive.org/dacs/EGAC00001000361).
