# Supplementary material for: Genomic ALK alterations in primary and relapsed neuroblastoma
Source: Br J Cancer. 2023 Feb 17;128(8):1559–71. doi: 10.1038/s41416-023-02208-y (PMC10070426; doi:10.1038/s41416-023-02208-y)
Supplement: Supplementary file 1 — Supplementary Material [file 41416_2023_2208_MOESM1_ESM.pdf]

# Genomic *ALK* alterations in primary and relapsed neuroblastoma

Carolina Rosswog, Jana Fassunke, Angela Ernst, Birgid Schömig-Markiefka, Sabine Merkelbach-Bruse, Christoph Bartenhagen, Maria Cartolano, Sandra Ackermann, Jessica Theissen, Mirjam Blattner-Johnson, Barbara Jones, Kathrin Schramm, Janine Altmüller, Peter Nürnberg, Monika Ortmann, Frank Berthold, Martin Peifer, Reinhard Büttner, Frank Westermann, Johannes H. Schulte, Thorsten Simon, Barbara Hero, Matthias Fischer

**Correspondence to:**

**matthias.fischer@uk-koeln.de**

**Supplementary Information**

Supplementary Figure 1

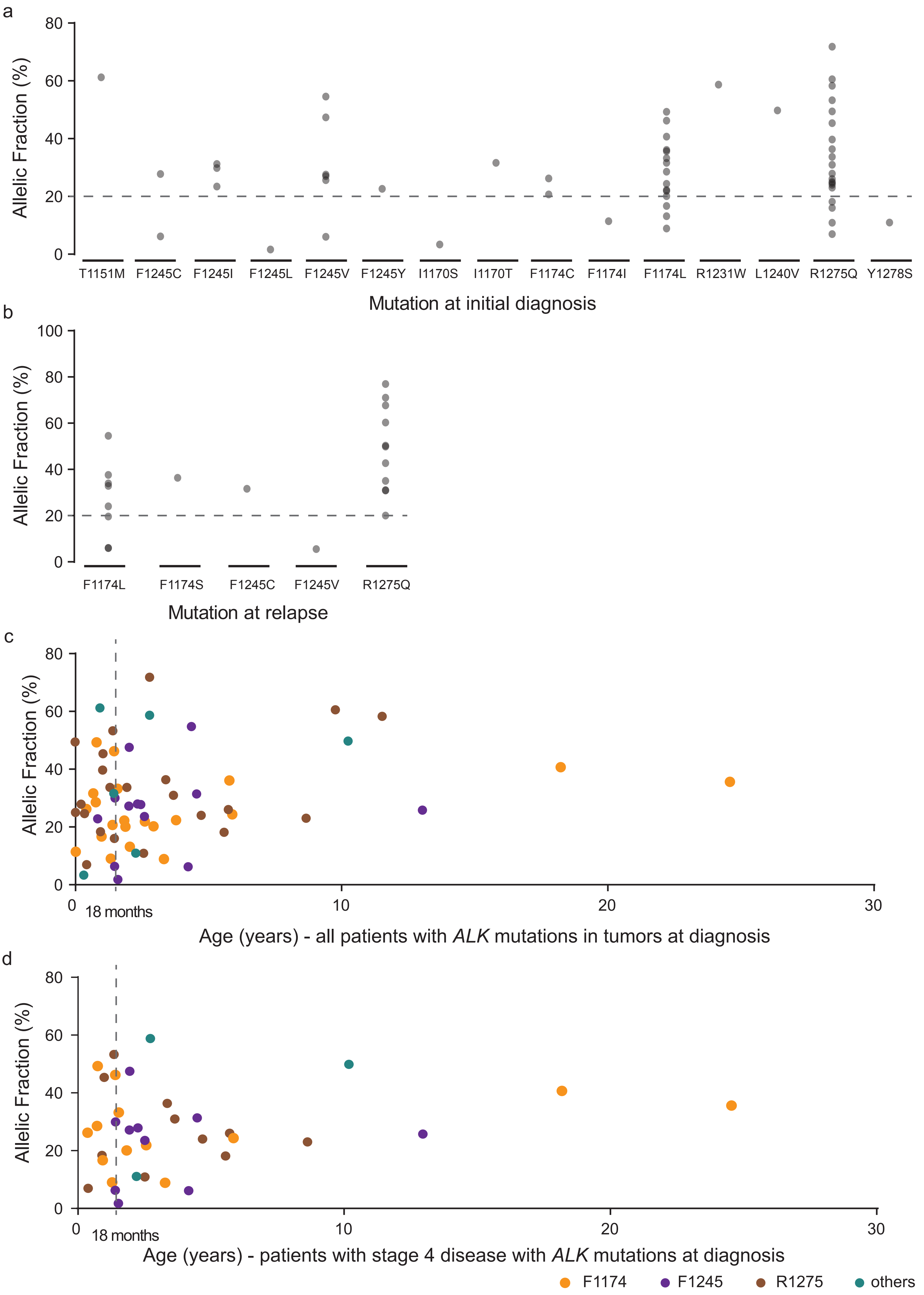

**Supplementary Figure 1. Allelic fractions of *ALK* mutations detected in neuroblastoma samples.**

(a) Allelic fractions of *ALK* mutations detected in neuroblastoma samples at initial diagnosis. (b) Allelic fractions of *ALK* mutations detected in neuroblastoma samples at relapse. (c) Allelic fractions of *ALK* mutations detected in neuroblastoma samples at diagnosis in dependence of patients' age at diagnosis. (d) Allelic fractions of *ALK* mutations detected in stage 4 neuroblastoma samples at diagnosis in dependence of patients' age at diagnosis.

## Supplementary Figure 2

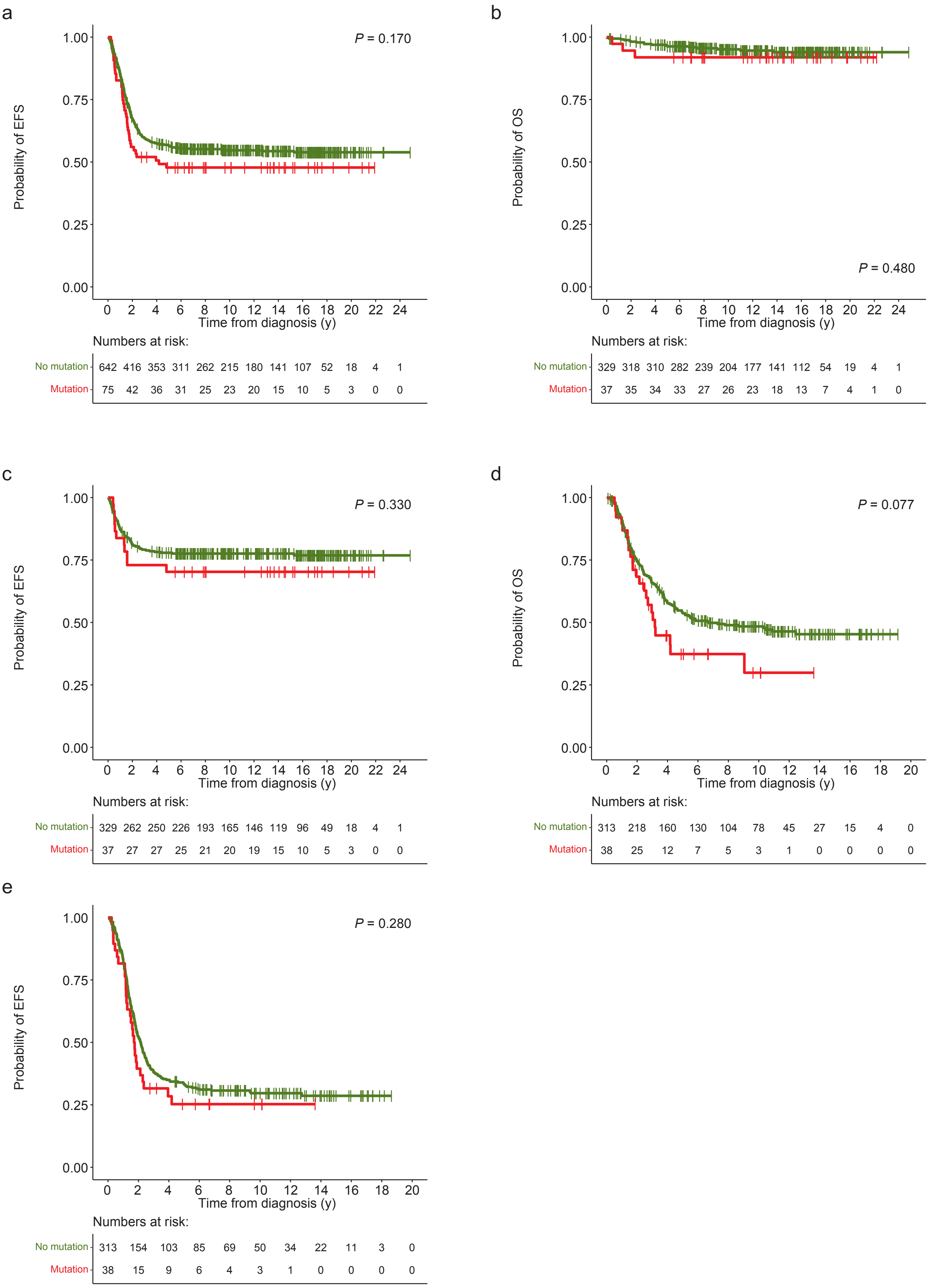

**Supplementary Figure 2. Impact of *ALK* mutations detected in neuroblastoma at diagnosis on patients' outcome.**

(a) EFS of patients with *ALK*-mutated tumors *versus* patients with *ALK* non-mutated tumors at diagnosis (5-year EFS, 48% *versus* 57%). (b) OS of non-high-risk patients with *ALK*-mutated *versus* non-mutated tumors at diagnosis (5-year OS, 92% *versus* 96%). (c) EFS of non-high-risk patients with *ALK*-mutated *versus* non-mutated tumors at diagnosis (5-year EFS, 70% *versus* 78%). (d) OS of high-risk patients with *ALK*-mutated *versus* non-mutated tumors at diagnosis (5-year OS, 37% *versus* 54%). (e) EFS of high-risk patients with *ALK*-mutated *versus* non-mutated tumors at diagnosis (5-year EFS, 25% *versus* 33%). *P* values were calculated by log-rank and, in case of non-proportional hazards, Gehan-Breslow test. OS, overall survival; EFS event-free-survival; y, years.

Supplementary Figure 3

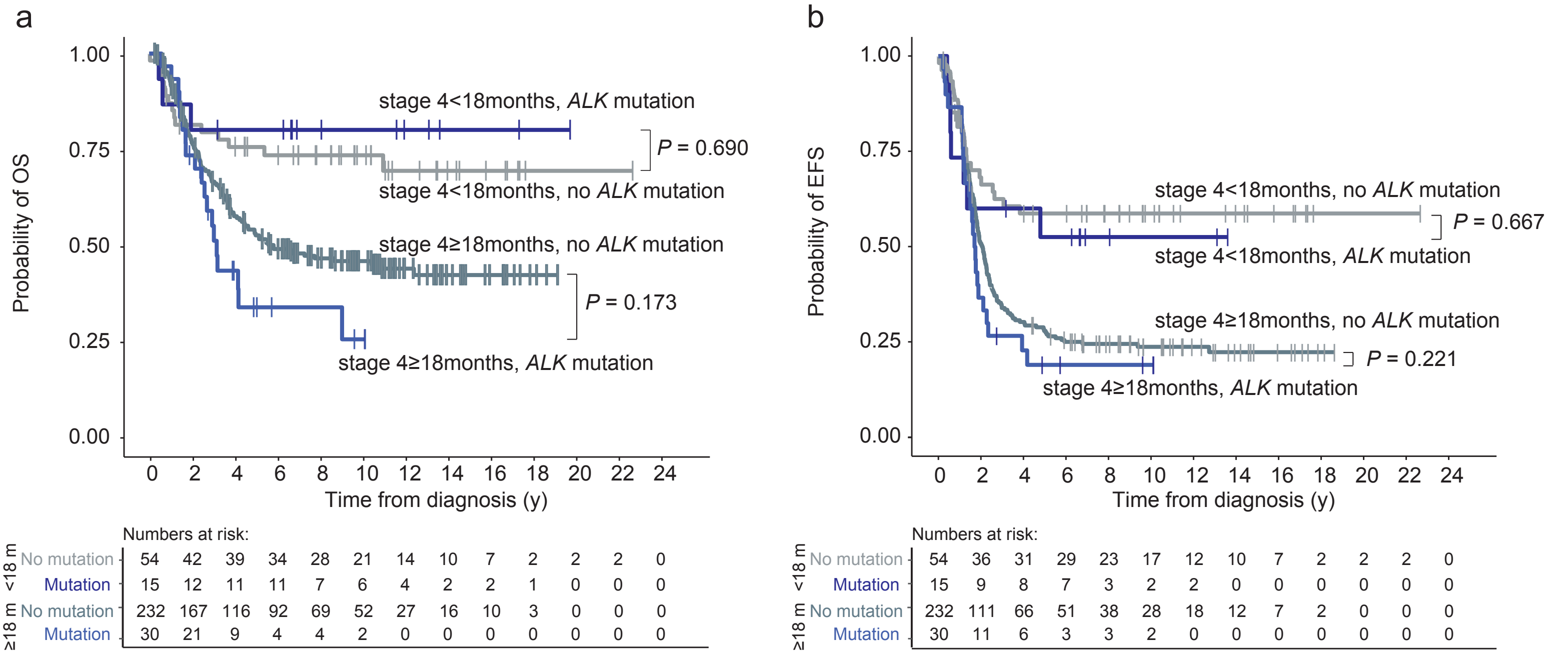

**Supplementary Figure 3. Impact of *ALK* mutations on survival of patients with stage 4 neuroblastoma.**

(a) OS of patients <18 months of age with stage 4 tumors harboring *ALK* mutations versus tumors lacking *ALK* mutations (5-year OS: 80% *versus* 76%). OS of patients ≥18 months of age with stage 4 tumors harboring *ALK* mutations versus tumors lacking *ALK* mutations (5-year OS: 34% *versus* 52%). (b) EFS of patients <18 months of age with stage 4 tumors harboring *ALK* mutations versus tumors lacking *ALK* mutations (5-year EFS: 53% *versus* 59%). OS of patients ≥18 months of age with stage 4 tumors harboring *ALK* mutations versus tumors lacking *ALK* mutations (5-year EFS: 19% *versus* 28%). *P* values were calculated by log-rank and, in case of non-proportional hazards, Gehan-Breslow test. OS, overall survival; EFS event-free-survival; y, years.

# Supplementary Figure 4

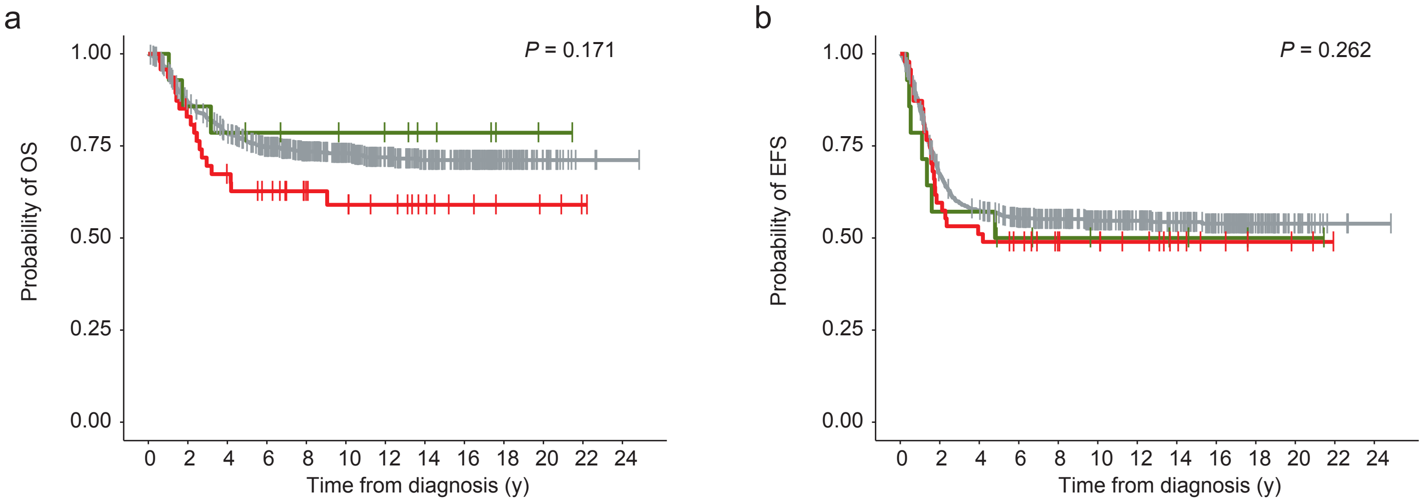


a — Numbers at risk:

| | | | | | | | | | | | | | |
|---|---|---|---|---|---|---|---|---|---|---|---|---|---|
| No mutation | 642 | 536 | 470 | 412 | 343 | 282 | 222 | 168 | 127 | 58 | 19 | 4 | 1 |
| AF≤20 | 14 | 12 | 11 | 10 | 9 | 8 | 7 | 5 | 4 | 2 | 1 | 0 | 0 |
| AF>20 | 47 | 38 | 29 | 25 | 18 | 16 | 13 | 9 | 6 | 4 | 3 | 1 | 0 |

b — Numbers at risk:

| | | | | | | | | | | | | | |
|---|---|---|---|---|---|---|---|---|---|---|---|---|---|
| No mutation | 642 | 416 | 353 | 311 | 262 | 215 | 180 | 141 | 107 | 52 | 18 | 4 | 1 |
| AF≤20 | 14 | 8 | 8 | 6 | 5 | 4 | 4 | 3 | 2 | 1 | 1 | 0 | 0 |
| AF>20 | 47 | 28 | 24 | 21 | 16 | 15 | 12 | 8 | 5 | 3 | 2 | 0 | 0 |

**Supplementary Figure 4. Impact of cloncal *versus* subclonal *ALK* mutations detected in neuroblastoma at diagnosis on patients' outcome.**

(a) OS of neuroblastoma patients with tumors harboring no *ALK* mutation *versus ALK* mutations at allelic fractions ≤20% *versus ALK* mutations at allelic fractions >20% (5-year OS 76% *versus* 79% *versus* 63%). (b) EFS of neuroblastoma patients with tumors harboring no *ALK* mutation *versus ALK* mutations at allelic fractions ≤20% *versus ALK* mutations at allelic fractions >20% (5-year EFS 57% *versus* 50% *versus* 49%). *P* values were calculated by log-rank. OS, overall survival; EFS, event-free-survival; y, years; AF, allelic fraction.

Supplementary Figure 5

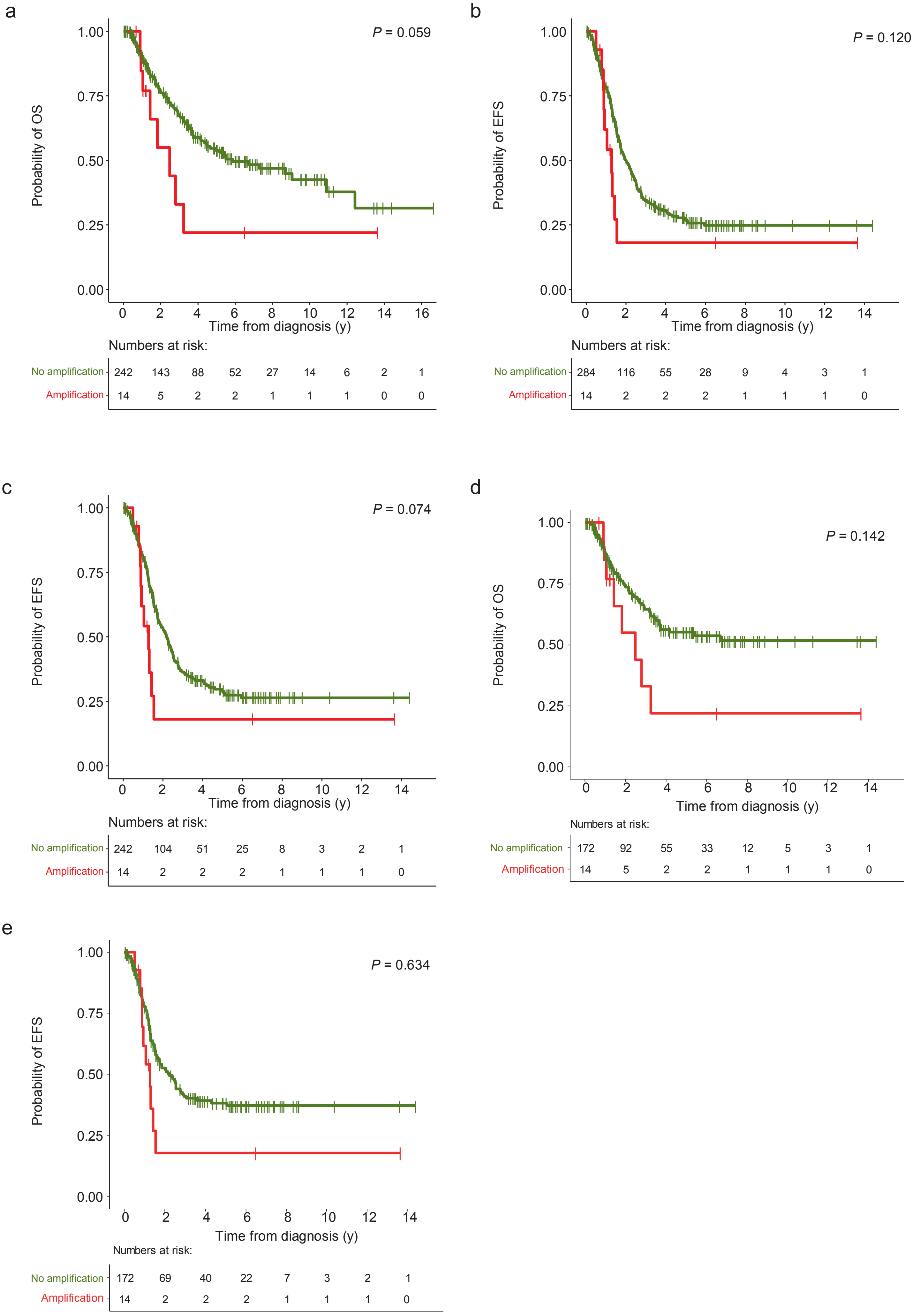

**Supplementary Figure 5. Impact of *ALK* amplifications detected in neuroblastoma at diagnosis on patients' outcome.**

(a) OS of high-risk patients with *ALK*-amplified tumors *versus* patients with non-amplified tumors at diagnosis (5-year OS, 22% *versus* 54%). (b) EFS of patients with *ALK*-amplified tumors *versus* non-amplified tumors at diagnosis (5-year EFS, 18% *versus* 27%). (c) EFS of high-risk patients with *ALK*-amplified *versus* non-amplified tumors at diagnosis (5-year EFS, 18% *versus* 29%). (d) OS of patients with *MYCN*-amplified tumors with additional *ALK* amplification *versus* tumors without *ALK* amplification at diagnosis (5-year OS 22% *versus* 55%). (e) EFS of patients with *MYCN*-amplified tumors with additional *ALK* amplification *versus* tumors without *ALK* amplification at diagnosis (5-year-EFS 18% *versus* 38%). *P* values were calculated by log-rank and, in case of non-proportional hazards, Gehan-Breslow test. OS, overall survival; EFS event-free-survival; y, years.

# Supplementary Figure 6

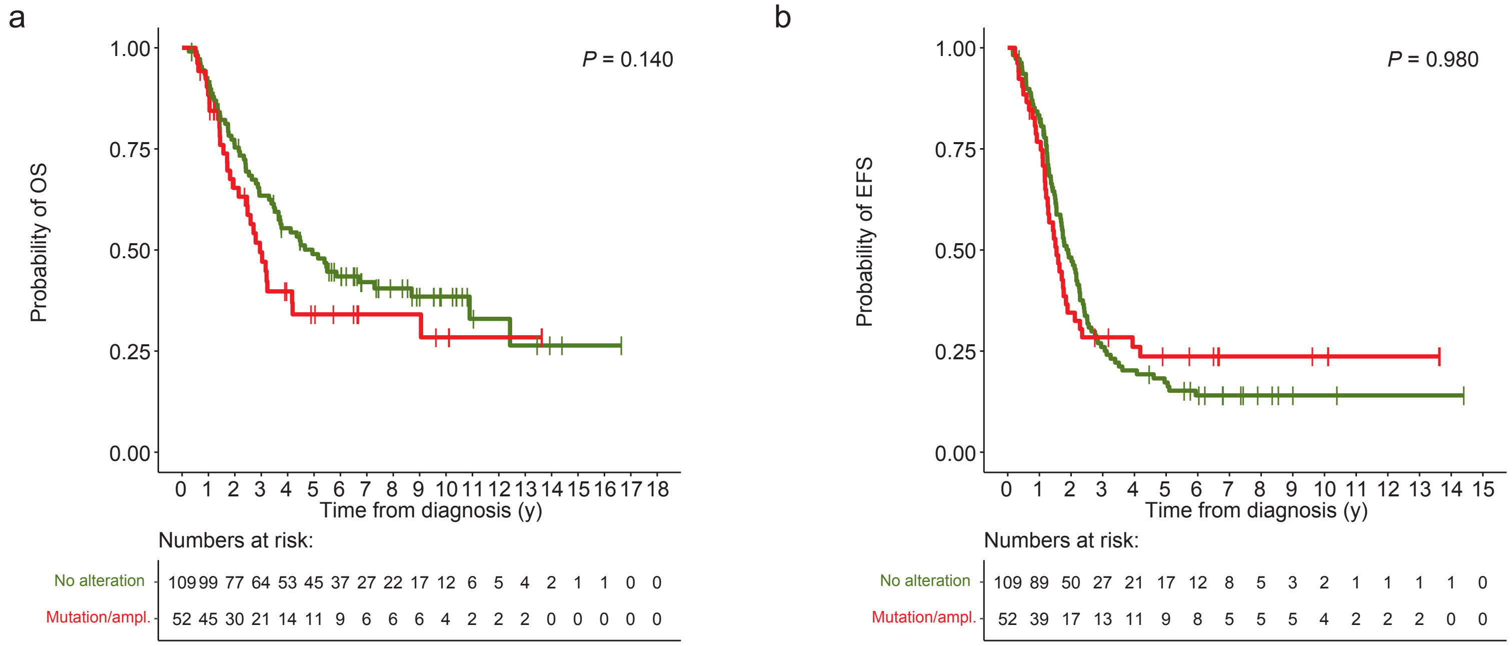

**Supplementary Figure 6. Impact of *ALK* alterations detected in neuroblastoma at diagnosis on patients' outcome.**

(a) OS of high-risk patients with *ALK*-altered *versus* non-altered tumors at diagnosis (5-year OS, 34% *versus* 49%). (b) EFS of high-risk patients with *ALK*-altered *versus* non-altered tumors at diagnosis (5-year EFS, 17% *versus* 24%). *P* values were calculated by log-rank and, in case of non-proportional hazards, Gehan-Breslow test. OS, overall survival; EFS, event-free-survival; y, years; ampl, amplification.

Supplementary Figure 7

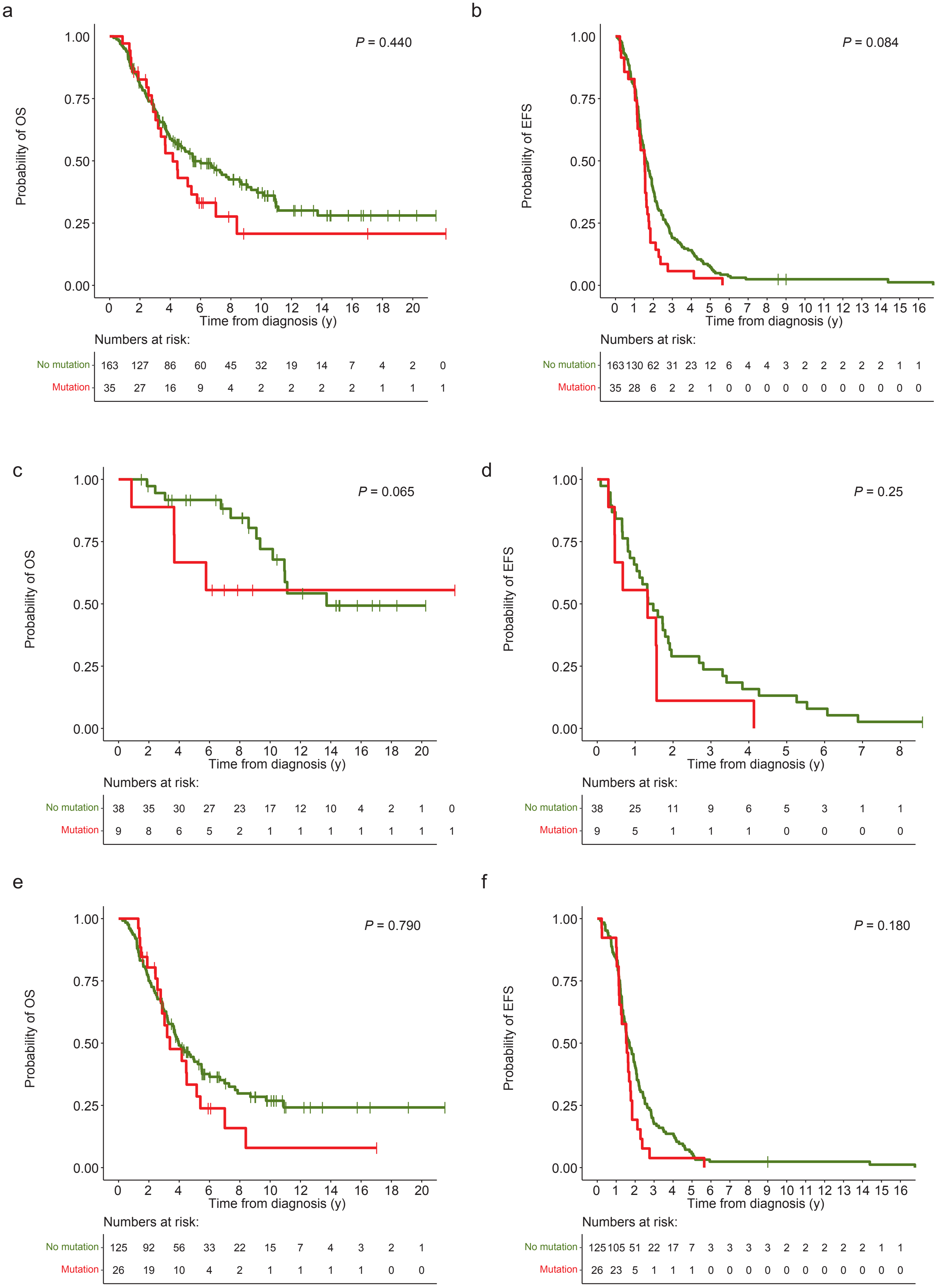

**Supplementary Figure 7. Impact of *ALK* mutations detected in relapsed neuroblastoma on patients' outcome.**

(a) OS of patients with *ALK*-mutated tumors *versus* patients with non-mutated tumors at relapse (5-year OS, 43% *versus* 54%). (b) EFS of patients with *ALK*-mutated tumors *versus* patients with non-mutated tumors at relapse (5-year EFS, 3% *versus* 7%). (c) OS of non-high-risk patients with *ALK*-mutated *versus* non-mutated tumors at relapse (5-year OS, 67% *versus* 92%). (d) EFS of non-high-risk patients with *ALK*-mutated *versus* non-mutated tumors at relapse (5-year EFS, 0% *versus* 13%). (e) OS of high-risk patients with *ALK*-mutated *versus* non-mutated tumors at relapse (5-year OS, 33% *versus* 43%). (f) EFS of high-risk patients with *ALK*-mutated *versus* non-mutated tumors at relapse (5-year EFS, 4% *versus* 6%). *P* values were calculated by log-rank and, in case of non-proportional hazards, Gehan-Breslow test. OS, overall survival; EFS, event-free-survival; y, years.

Supplementary Figure 8

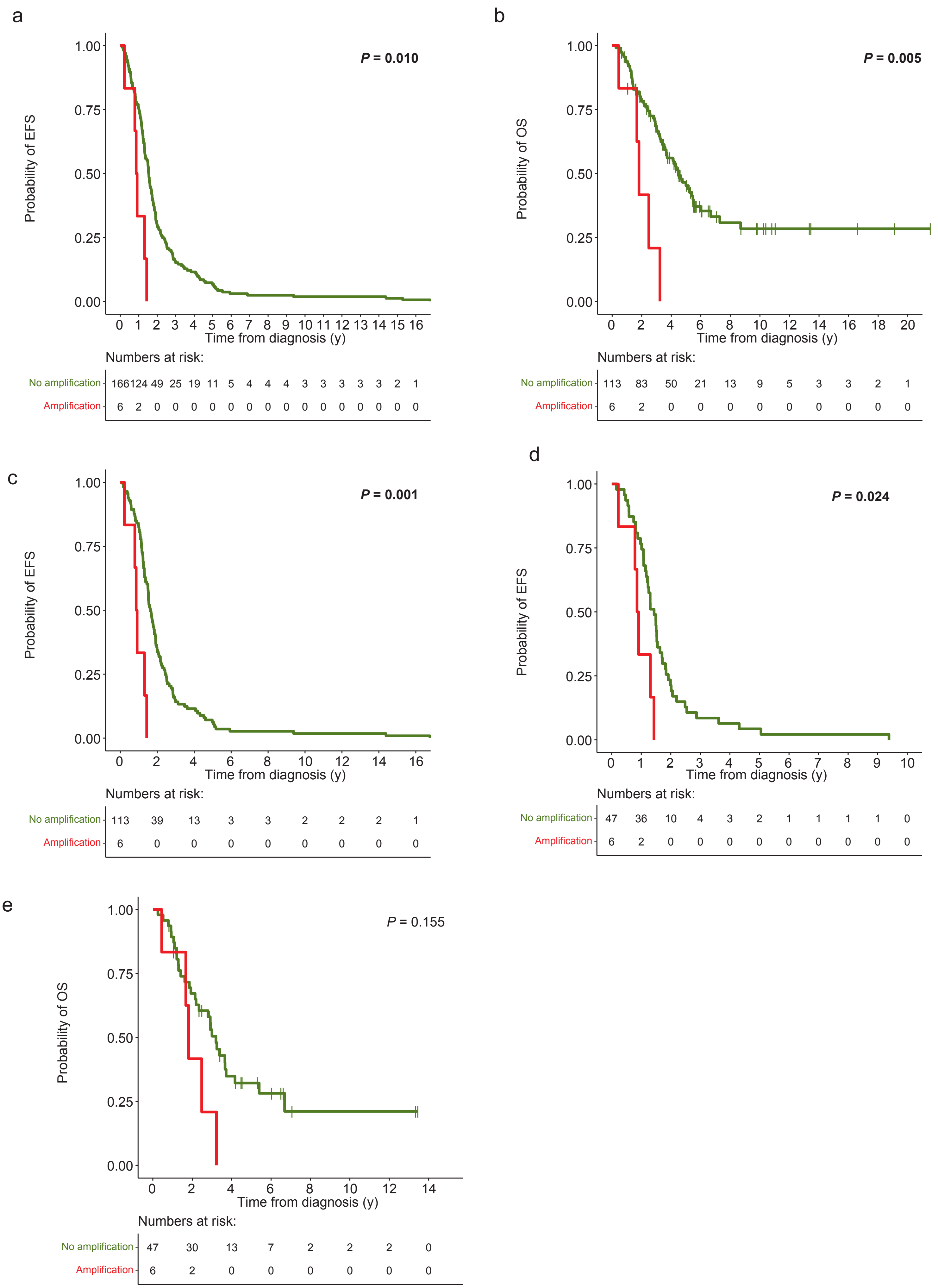

**Supplementary Figure 8. Impact of *ALK* amplifications detected in relapsed neuroblastoma on patients' outcome.**

(a) EFS of patients with *ALK*-amplified tumors *versus* patients with non-amplified tumors at relapse (5-year EFS, 0% *versus* 7%). (b) OS of high-risk patients with *ALK*-amplified *versus* non-amplified tumors at relapse (5-year OS, 0% *versus* 45%). (c) EFS of high-risk patients with *ALK*-amplified *versus* non-amplified tumors at relapse (5-year EFS, 0% *versus* 6%). (d) EFS of patients with *MYCN*-amplified tumors showing co-amplification of *ALK versus* no co-amplification of *ALK* at relapse (5-year EFS, 0% versus 4%). (e) OS of patients with *MYCN*-amplified tumors with co-amplification of *ALK versus* no co-amplification of *ALK* at relapse (5-year OS, 0% *versus* 32%). *P* values were calculated by log-rank and, in case of non-proportional hazards, Gehan-Breslow test. OS, overall survival; EFS, event-free-survival; y, years.

Supplementary Figure 9

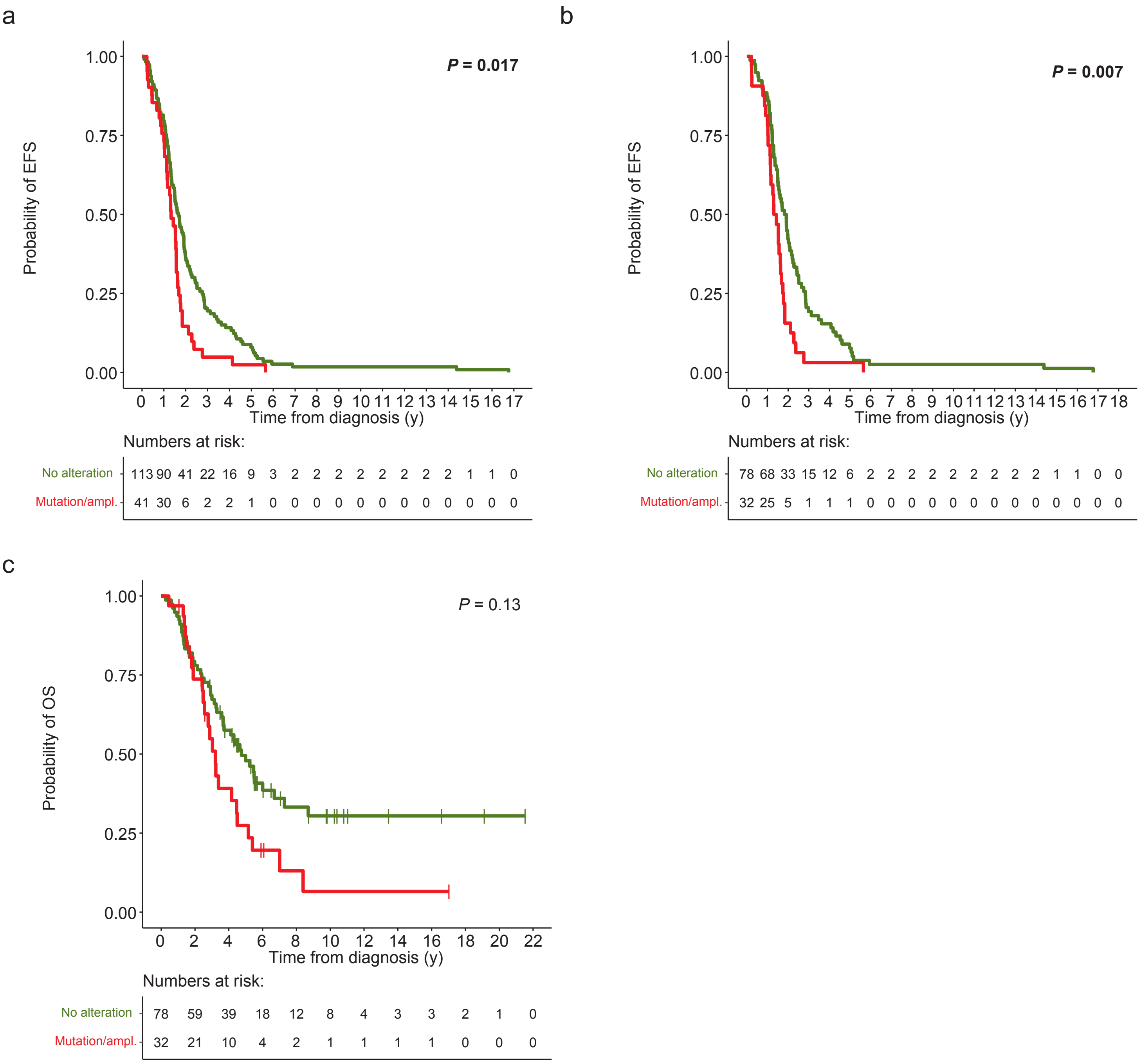

**Supplementary Figure 9. Impact of *ALK* alterations detected in relapsed neuroblastoma on patients' outcome.**

(a) EFS of patients with *ALK*-altered *versus* non-altered tumors at relapse (5-year EFS, 2% *versus* 8%). (b) EFS of high-risk patients with *ALK*-altered *versus* non-altered tumors at relapse (5-year EFS, 3% *versus* 8%). (c) OS of high-risk patients with *ALK*-altered *versus* non-altered tumors at relapse (5-year OS, 34% *versus* 49%). *P* values were calculated by log-rank and, in case of non-proportional hazards, Gehan-Breslow test. OS, overall survival; EFS, event-free-survival; y, years; ampl, amplification.

Supplementary Figure 10

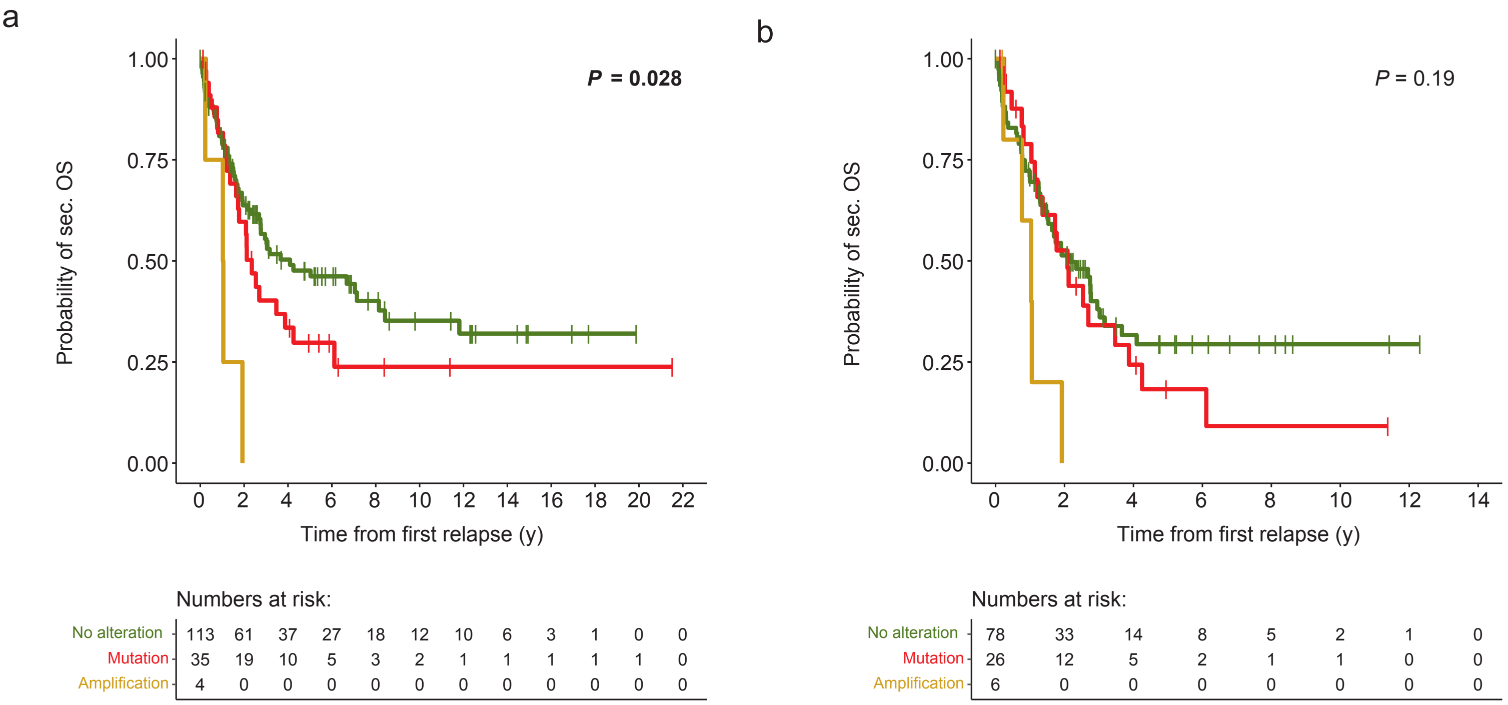

**Supplementary Figure 10. Secondary overall survival of patients with tumors harboring *ALK* alterations versus no *ALK* alterations.**

(a) Secondary OS of patients with *ALK* mutation *versus* *ALK* amplification *versus* *ALK* non-altered tumors calculated from time of relapse (5-year OS, 30% *versus* 0% *versus* 48%). (b) Secondary OS of high-risk patients with *ALK*-amplified *versus* *ALK*-mutated tumors *versus* tumors without *ALK* alteration (5-year OS, 0% *versus* 18% *versus* 29%). *P* values were calculated by log-rank and, in case of non-proportional hazards, Gehan-Breslow test. Sec. OS, secondary overall survival; y, years.

Supplementary Figure 11

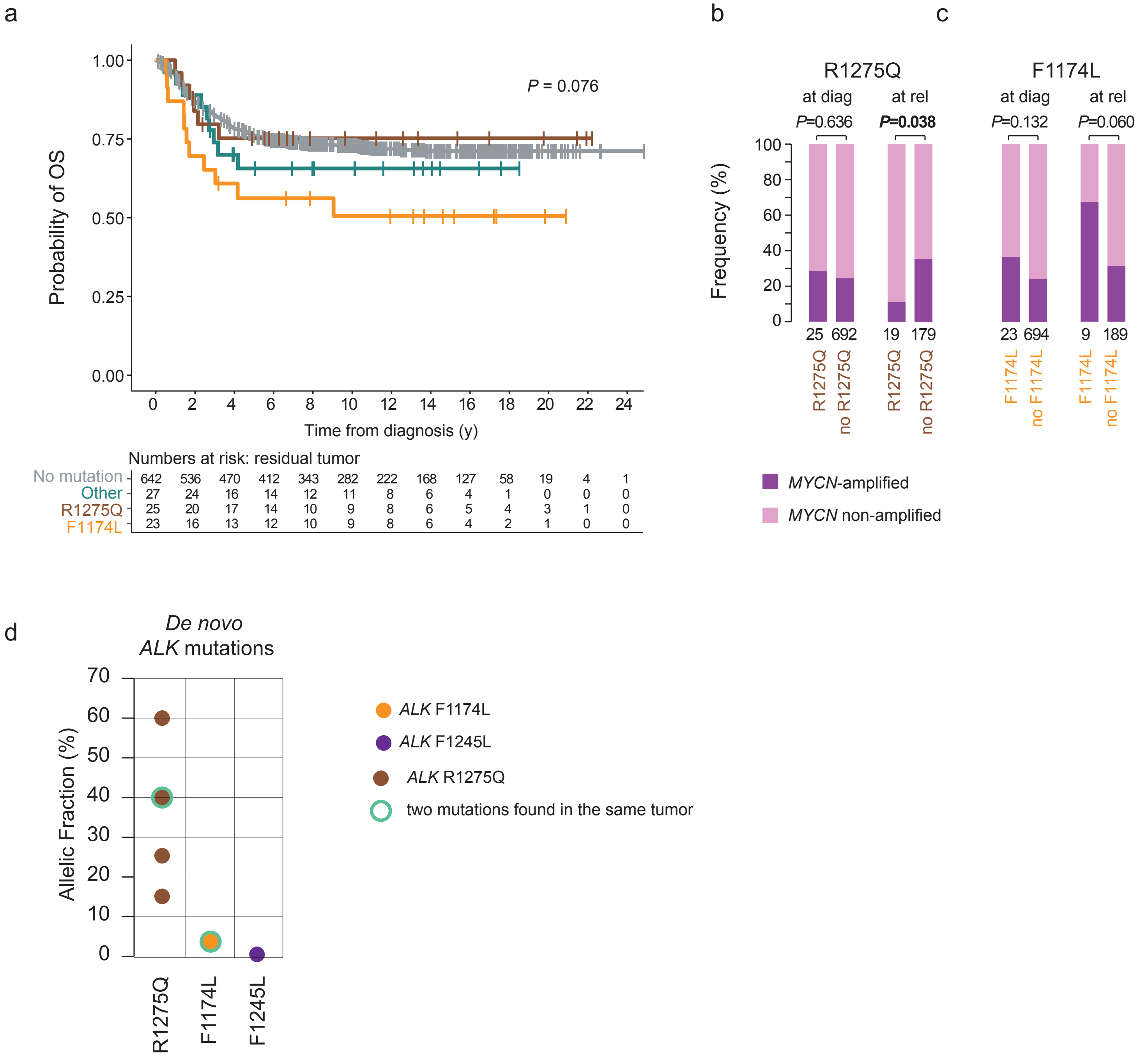

**Supplementary Figure 11. Characteristics of *ALK* mutation type.**

(a) OS of patients with F1174L-mutated *versus* R1275Q-mutated *versus* other *ALK* mutations *versus* non-mutated tumors at diagnosis (5-year OS, 56% *versus* 75% *versus* 66% *versus* 76%). *P* value was calculated by log-rank, OS, overall survival; y, years. (b) Frequencies of *MYCN* amplification in R1275Q-mutated *versus* non-mutated tumors at diagnosis and at relapse. At diag, at diagnosis; at rel, at relapse. (c) Frequencies of *MYCN* amplification in F1174L-mutated *versus* non-mutated tumors at diagnosis and at relapse. (d) Allelic fractions as determined by ddPCR and type of *de novo* mutations in neuroblastoma samples at relapse. Circles indicate two distinct *de novo* mutations that were detected in the same tumor. Allelic fractions refer to the time point of first occurrence in case of information on multiple time points. Two of eight *de novo* mutations (F1174C and F1245C) are not shown in the graph, as they were analyzed by dideoxy-sequencing only, and allelic fractions were thus not available.

**Supplementary Table 1. Details on neuroblastoma patients included in the study.**

F, female; M, male; NHR, non-high-risk; HR, high-risk; amp, amplification; non amp, no amplification; Panel, panel next-generation sequencing; Seq, next-generation sequencing; Sanger, dideoxy sequencing; FISH, fluorescence *in situ* hybridization; Inform, whole-exome sequencing data provided by the INFORM program; CT, chemotherapy according to non-high risk protocol; HR-CT, chemotherapy according to high-risk protocols; no CT, no chemotherapy.

**Supplementary Table 2. Primer pairs used for dideoxy sequencing.**
